# Polymer Brush‐Based Asymmetric Porous Patch with Excellent Anti‐Bacterial and Anti‐Contamination Properties Enables Infectious Abdominal Wall Defect Repair

**DOI:** 10.1002/advs.202500865

**Published:** 2025-04-07

**Authors:** Zeping Huang, Weiwen Liang, Binying Peng, Yang Ouyang, Zixin Chen, Mawsoom Mamut, Zhipeng Huang, Hui Wang, Rongkang Huang, Bo Ma, Bingna Zheng, Jian Cai

**Affiliations:** ^1^ Department of General Surgery (Colorectal Surgery) Guangdong Institute of Gastroenterology Biomedical Innovation Center Guangdong Provincial Key Laboratory of Colorectal and Pelvic Floor Diseases The Sixth Affiliated Hospital Sun Yat‐sen University Guangzhou 510655 P. R. China; ^2^ Department of Urology The First People's Hospital of Kashi Kashi 844099 P. R. China; ^3^ Department of Urology The Sixth Affiliated Hospital Sun Yat‐sen University Guangzhou 510655 P. R. China; ^4^ The Eighth Affiliated Hospital Sun Yat‐sen University Shenzhen 518000 P. R. China; ^5^ Department of Colorectal Surgery Shenzhen Second People's Hospital First Affiliated Hospital of Shenzhen University Medical Innovation Technology Transformation Center of Shenzhen Second People's Hospital Shenzhen University Shenzhen 518035 P. R. China

**Keywords:** anti‐bacterial, anti‐contamination, asymmetric porous patch, infectious abdominal wall defect, zwitterionic polyvinyl alcohol molecular brush

## Abstract

Well‐integration of multiple bioactive components into a biological patch without damaging its cellular matrix structure is crucial for infectious abdominal wall defect repair but remains challenging. Herein, a novel asymmetric biological composite patch (bPVA/SIS^+^‐NP) is developed by in situ introducing zwitterionic polyvinyl alcohol molecular brush (bPVA) hydrogel to a cationic small intestinal submucosal decellularized matrix (SIS^+^) via a self‐induced phase separation based united strategy. Due to its high hydrogen bonding crosslinking and strong mechanical interlocking with SIS^+^‐NP layer, bPVA layer can maintain stable anti‐contamination and the corresponding anti‐adhesion properties in the contaminated environment. On the basis of preserving an original extracellular matrix skeleton, SIS^+^‐NP layer can show strong contact anti‐bacterial ability at the acute stage of repair together with the prolonged drug release during the healing stage of repair. Furthermore, the in situ combination of bPVA and SIS^+^‐NP layers can lead to a high and stable burst pressure tolerance. By comprehensive control of bacteria and their necrotic products, bPVA/SIS^+^‐NP patch can achieve anti‐infection, anti‐adhesion, and pro‐healing properties in the infectious abdominal wall defect on rats. Therefore, the bPVA/SIS^+^‐NP patch opens a new avenue for bio‐friendly construction of multifunctional biological patches to address the stringent requirements of infectious abdominal wall defect repair.

## Graphical abstract

A zwitterionic asymmetric patch is developed via self‐induced phase separation. The cationic layer exhibits anti‐bacterial efficacy and releases pro‐healing factor, while the zwitterionic hydrogel prevents contamination and visceral adhesion. The strong mechanical interlocking of two layers leads to a high and stable burst pressure tolerance. This composite patch can regulate the infectious microenvironment and demonstrates therapeutic potential for repairing infectious abdominal wall defects.

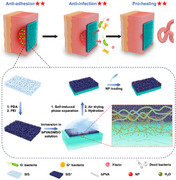

## 1 Introduction

**Figure 1:**
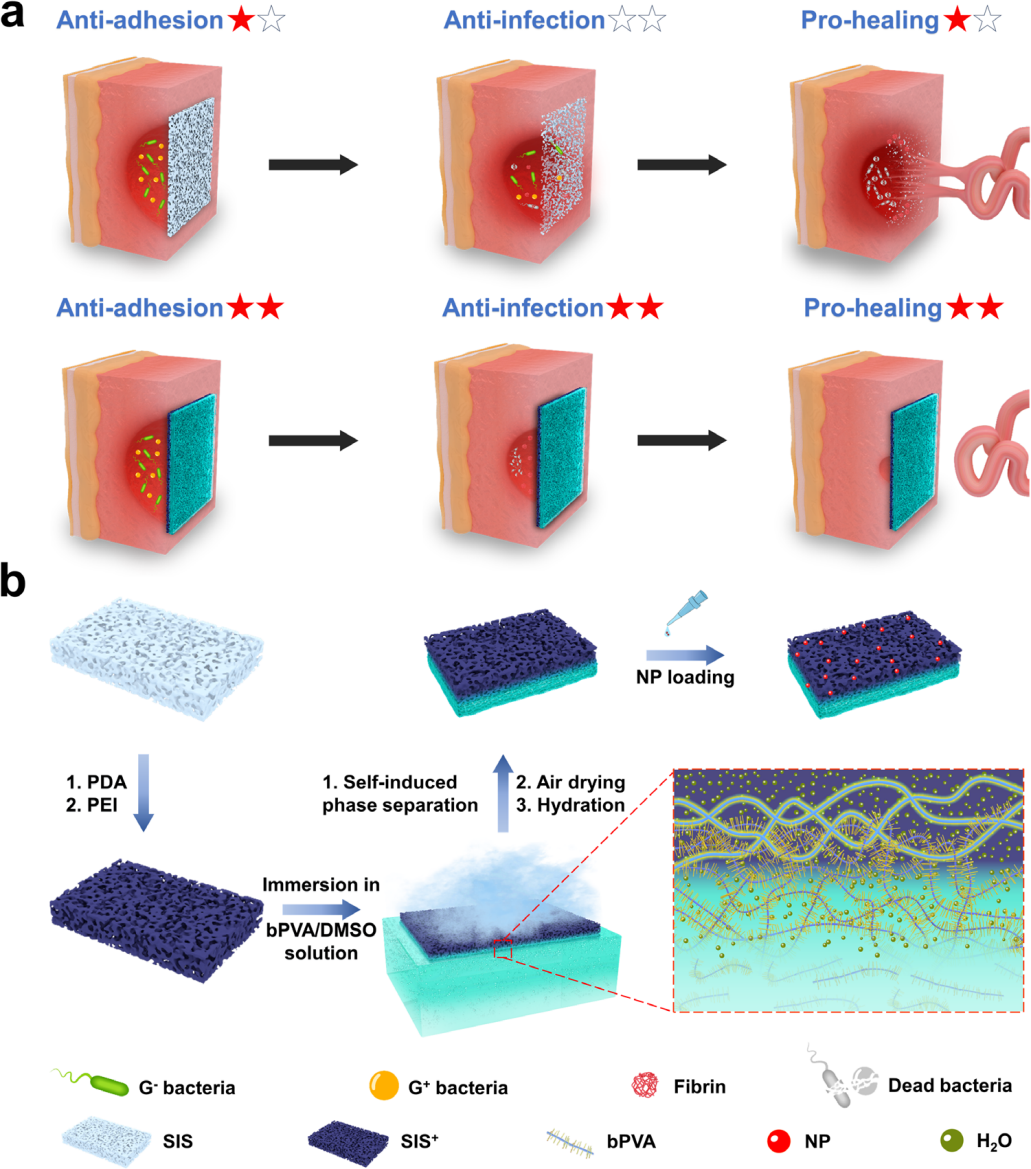
Preparation and structure of bPVA/SIS^+^‐NP patch, and tension‐free repair of infectious abdominal wall defect. a) Schematic illustrations of conventional biological patch and our bPVA/SIS^+^‐NP patch used for tension‐free repair. Conventional biological patch degrades rapidly and lacks anti‐infection properties, leading to mechanical instability, visceral adhesion, and poor defect healing in an infectious abdominal microenvironment. Our asymmetric bPVA/SIS^+^‐NP patch shows prolonged mechanical stability, excellent anti‐bacterial activities, and anti‐contamination properties, thus effectively controlling infection to prevent visceral adhesion and accelerate the healing process of infectious abdominal wall defect. b) The procedure of constructing bPVA/SIS^+^‐NP patch (the inset: schematic illustration of the asymmetric porous structure).

Infectious abdominal wall defect faces the challenge of complex contamination microenvironment that poses significant difficulties in treatment.^[^ ^1^, ^2^, ^3^, ^4^ ^]^ Commercial biological patches with anti‐adhesion property and certain tissue reconstruction capability, such as small intestinal submucosal decellularized matrix (SIS) and basic membrane (BM), are considered as one of the most promising repair materials for infectious abdominal wall defect.^[^ ^5^, ^6^, ^7^, ^8^, ^9^, ^10^ ^]^ However, biological patches exhibit limitations in infection control and are prone to rapid degradation in vivo, leading to a significant decrease of mechanical strength,^[^ ^11^ ^]^ thus resulting in significant postoperative complications such as local abscess, secondary abdominal adhesion, and high recurrent rate (**Figure** **1**a).^[^ ^12^, ^13^ ^]^ Therefore, on the premise of ensuring the tissue reconstruction capability, the preparation of biocompatible patches with excellent anti‐infection ability and anti‐adhesion property is essential to the development of novel repair materials for infectious abdominal wall defect.

Unlike superficial tissue damage, such as skin wounds, the microenvironment of an infectious abdominal wall defect represents a complex system typically resulting from multiple factors.^[^ ^14^, ^15^ ^]^ It is crucial to impart diverse bioactivities to biological patches, including anti‐bacterial ability, anti‐adhesion capability, and pro‐healing activity.^[^ ^16^, ^17^, ^18^ ^]^ Moreover, the degradation should also be well‐controlled to provide consistent mechanical support throughout the tissue reconstruction process. In general, the repair of infectious abdominal soft tissue requires implant materials to have excellent anti‐bacterial ability; meanwhile, the complicated abdominal microenvironment demands implant materials to maintain excellent biocompatibility and present anti‐adhesion capability. Despite considerable efforts dedicated to this area, rare patches could simultaneously achieve these contradictory properties. Many studies usually focus on enhancing anti‐bacterial bioactivity of biomaterials,^[^ ^19^, ^20^, ^21^, ^22^ ^]^ but their anti‐bacterial components may lead to cell toxicity^[^ ^23^, ^24^ ^]^ or even secondary visceral adhesion. For example, the metabolic activity and subsequent death of bacteria during anti‐bacterial processes will initiate the release of inflammatory adhesion factors, promote fibrin deposition, and facilitate biofilm formation, ultimately resulting in abdominal adhesion and impaired healing.^[^ ^25^ ^]^ As for the preparation of high‐performance patches, traditional surface chemical modification strategies often lead to collagen denaturation, adversely affecting their chemical and physical structures and thus impairing their resistance to infection; simple compositional strategies usually result in weak interfacial interactions among various functional components, hindering the synergistic realization of multiple bioactivities.^[^ ^26^, ^27^, ^28^, ^29^ ^]^ Therefore, the key to develop high‐performance biological patches with excellent anti‐bacterial and anti‐contamination properties lies in how to hierarchically assemble corresponding components without damaging the natural structure of biological patches.

Herein, a double‐layer asymmetric biological patch is obtained for infectious abdominal wall defect repair under the premise of preservation of natural loosely porous structure of decellularized matrix layer by integrating cationic small intestinal submucosal decellularized matrix (SIS^+^) with zwitterionic polymer brush grafted PVA (bPVA) layer through self‐induced phase separation, air drying, hydration, and nicotinamide phosphoribosyltransferase (NP)^[^ ^30^ ^]^ loading (Figure 1b). The polydopamine (PDA) and polyethyleneimine (PEI) polycations coating of SIS^+^ is formed through oxidative self‐polymerization reaction to postpone its degradation and increase its contact anti‐bacterial performance. By the subsequent self‐induced phase separation process, as the aqueous solution of SIS^+^ diffuses downward into the bPVA/DMSO solution, the bPVA polymer chains are gradually induced to crosslink with dense hydrogen bonds at the interface between SIS^+^ and bPVA/DMSO solution, thus forming the strong mechanical interlocking between their interfaces without damaging the natural structure of SIS^+^. After air drying and hydration, the SIS^+^ is firmly compounded with bPVA hydrogel layer, which can prevent contamination of the killed bacteria and fibrin even in the presence of localized material damage by forming a dense hydration layer through electrostatic interactions. The introduction of NP further endows healing promoting bioactivity to the patch. Moreover, the in situ combination of bPVA and SIS^+^‐NP layers can result in a burst pressure of up to 759 mm Hg and long‐term mechanical stability during the repair process. bPVA/SIS^+^‐NP patch exhibits excellent anti‐infection, visceral anti‐adhesion, and pro‐healing properties in the repair of infectious abdominal wall defects on rats. This bio‐friendly assembly strategy of developing multifunctional biological patch without damaging the natural structure opens up a new avenue for the efficient repair of infectious abdominal wall defect.

## 2 Results and Discussion

**Figure 2:**
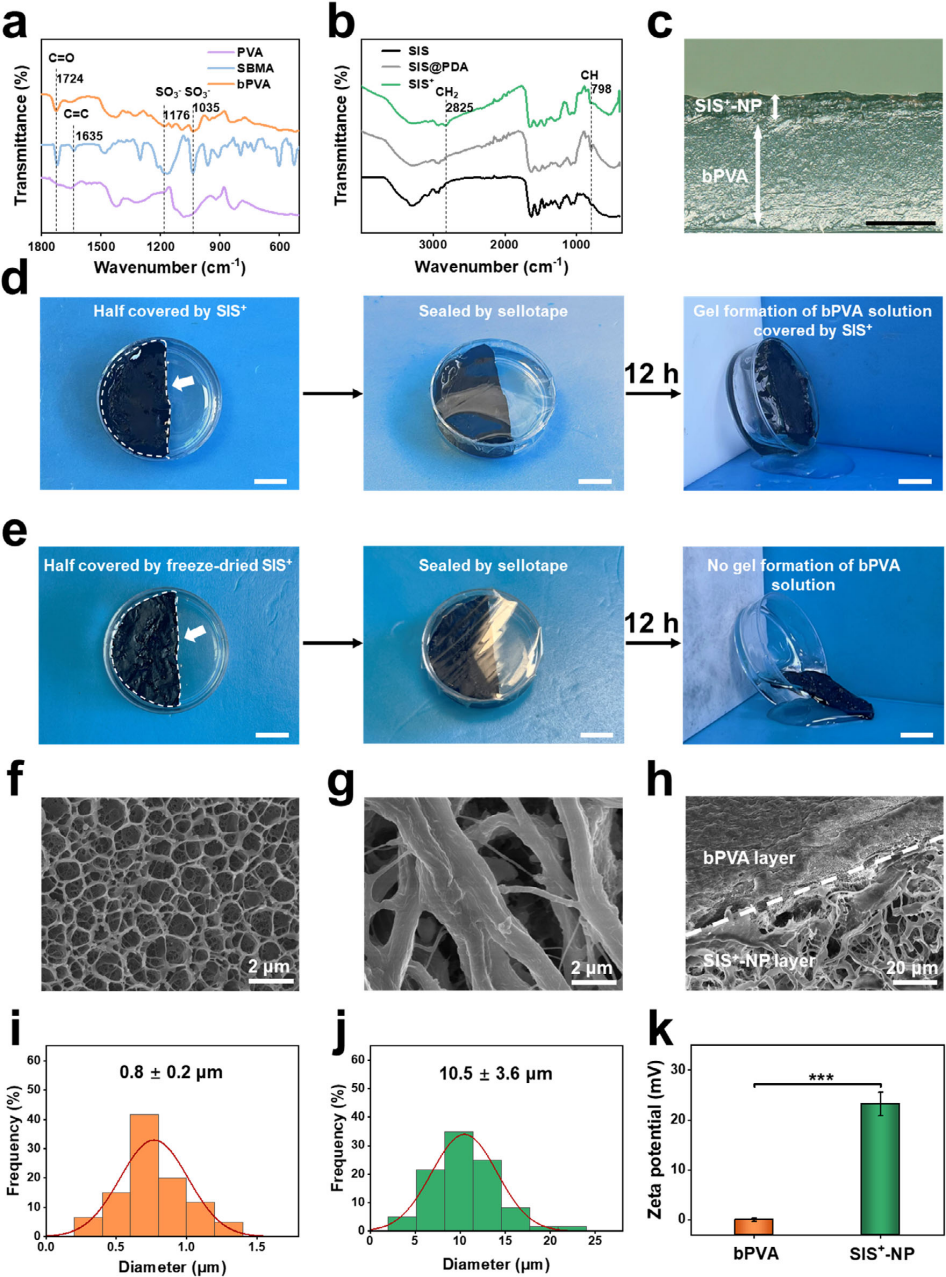
Asymmetric porous structure and physicochemical characterization of bPVA/SIS^+^‐NP patch. a) ATR‐FTIR spectra of PVA, SBMA, and bPVA. b) ATR‐FTIR spectra of SIS, SIS@PDA, and SIS^+^. c) Digital photo of the cross‐section of bPVA/SIS^+^‐NP patch. d,e) Digital photos of the gelation for bPVA/DMSO solution with (left) and without (right) the coverage of SIS^+^ (d) and freeze‐dried SIS^+^ (e) under the sealed condition with sellotape. f–h) SEM images of bPVA surface (f), SIS^+^‐NP surface (g), and the cross‐section of bPVA/SIS^+^‐NP patch (h). Scale bars: 0.5 mm in (c), 1 cm in (d, e). i,j) Pore size distributions on the surface of bPVA (i) and SIS^+^‐NP (j). k) Zeta potential of bPVA and SIS^+^‐NP. The data are the mean ± standard deviation (SD) (*n* = 3; *** *p* < 0.001).

To address the deficiency in visceral anti‐adhesion, PSBMA was grafted from PVA through free radical polymerization to obtain a kind of bPVA. Three kinds of bPVA with different grafting efficiencies (35.80%, 82.11%, and 99.04% for bPVA1, bPVA2, and bPVA3, respectively) were obtained by changing feeding weight ratios of PVA and SBMA (Figure S1, Supporting Information), and bPVA2 was finally chosen as the typical sample. Fourier transform infrared spectroscopy with attenuated total reflectance (ATR‐FTIR) is employed to investigate the structural changes from PVA to bPVA. Notably, the ATR‐FTIR spectroscopy of bPVA demonstrates new peaks at 1035 and 1176 cm^−1^, which can be ascribed to the stretching vibrational bands of sulfonate groups (S─O and S═O), and a new peak at 1724 cm^−1^ referring to the carbonyl group (C═O) stretching vibrations. The absence of the carbon‐carbon double bond (C═C) at ≈1635 cm^−1^, typically associated with the sulfobetaine methacrylate (SBMA) monomer, indirectly confirms the successful polymerization and grafting of PSBMA from PVA backbone (**Figure** **2**a). Proton nuclear magnetic resonance (^1^H‐NMR) spectroscopy validates the successful synthesis of the bPVA2, with a grafting efficiency of 81.2% according to the integral area of characteristic peaks (Figure S2, Supporting Information). Superhydrophilic PSBMA polymer hairs can effectively change the contact angle of PVA from 48.9° to 14.8° (Figure S3, Supporting Information).

To improve the anti‐bacterial and anti‐degradation properties of SIS, it is first functionalized by doping with PDA (SIS@PDA), evidenced by a color shift from white to black, together with a characteristic peak of substituted aromatic ring at 798 cm^−1^. Further modification with a 6 h immersion of PEI enhances the methylene peak at 2825 cm^−1^, as confirmed by ATR‐FTIR spectroscopy. These new peaks indicate the successful decoration of PEI onto the PDA layer, resulting in the cationic SIS@PDA‐PEI (SIS^+^) (Figure 2b). By facile covering the wet SIS^+^ on dimethyl sulfoxide (DMSO) solution of bPVA, gelation of bPVA chains happens during the self‐induced phase separation and a composite patch of bPVA/SIS^+^ can be obtained after air drying and hydration (Figure 2c). As shown in Figure 2d, during the self‐induced phase separation process, the aqueous solution in SIS^+^ diffuses downward into the bPVA/DMSO solution, and promotes the phase separation of bPVA chain to form dense hydrogen bond network at the interface with SIS^+^, thereby achieving strong mechanical interlocking of them without damaging the natural structure of SIS^+^. In contrast, the freeze‐dried SIS^+^ fails to trigger phase separation process of bPVA/DMSO solution (Figure 2e). After air drying and hydration, the final multifunctional bPVA/SIS^+^‐NP patch can be achieved. The thicknesses of bPVA hydrogel and SIS^+^‐NP layers are measured to be 791 ± 26 µm and 206 ± 11 µm, respectively (Figure S4, Supporting Information). As demonstrated by scanning electron microscopy (SEM), bPVA hydrogel layer exhibits a macroporous structure with an average pore size of 0.8 µm (Figure 2f,i). In contrast, SIS^+^‐NP layer retains the natural interconnected nanofibrous network of SIS, with a noticeably rougher surface due to the decoration of PDA‐PEI and NP (Figure 2g; Figure S5, Supporting Information). The mean pore diameter of SIS^+^‐NP is 10.5 µm (Figure 2j). The difference in pore diameter confirms the asymmetric porous structure of our bPVA/SIS^+^‐NP patch. As shown in the cross‐sectional SEM image, no obvious cleavage can be found at the interfacial region, which indicates that strong mechanical interlocking ensures a robust connection of the two layers (Figure 2h). Therefore, the sample can withstand a loading weight of 200 g in a lap‐shear test, and demonstrates an interfacial strength of 172 N m^−1^ in the 180‐degree peeling test (Figure S6, Supporting Information). With the different charge characteristics of the functional polymeric components, zeta potentials of bPVA and SIS^+^‐NP layers are 0 and 23.2 mV, respectively. bPVA is almost electrically neutral due to the zwitterionic PSBMA polymer hairs, while SIS^+^‐NP demonstrates obvious cationic surface property due to the coating of PEI (Figure 2k).

**Figure 3:**
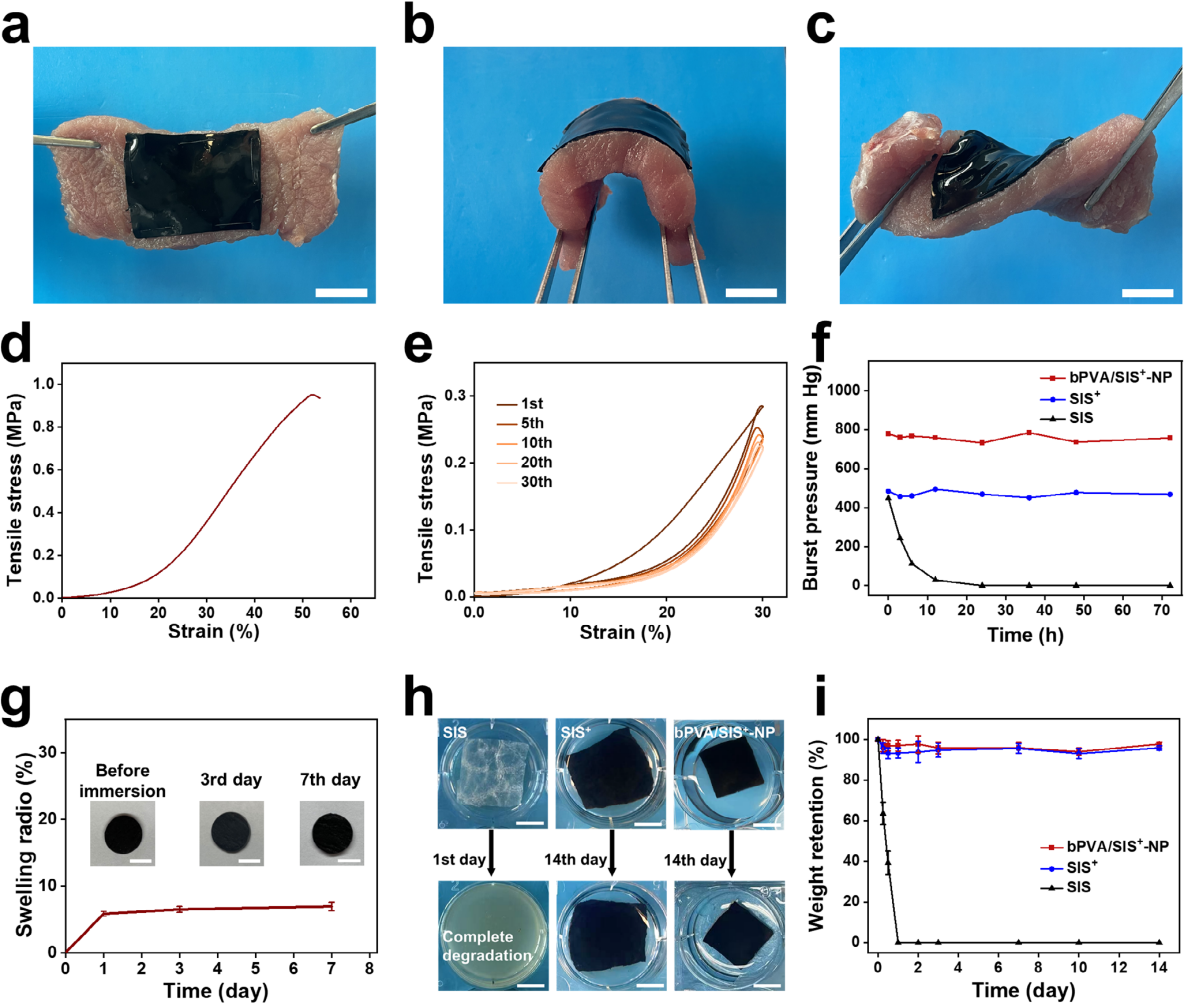
The mechanical, anti‐degradation, and anti‐swelling properties of bPVA/SIS^+^‐NP patch. a–c) Digital photos of bPVA/SIS^+^‐NP sutured onto porcine muscle, demonstrating its high flexibility. d) Tensile stress‐strain curve of bPVA/SIS^+^‐NP patch. e) 30% tensile loading−unloading curves of bPVA/SIS^+^‐NP patch. f) Burst pressure of bPVA/SIS^+^‐NP, SIS^+^, and SIS treated by 0.2 mg mL^−1^ collagenase I solution. g) Swelling ratios of bPVA/SIS^+^‐NP patch in PBS buffer. Insets: Digital photos of bPVA/SIS^+^‐NP before immersion and after immersion in PBS buffer for 3 and 7 days. h, i) Digital photos (h) and weight retention curves (i) for bPVA/SIS^+^‐NP, SIS^+^, and SIS in 0.2 mg mL^−1^ collagenase I solution. Scale bars: 3 cm in (a–c) and 1 cm in (g, h). The data are the mean ± SD (*n* = 3).

The ideal patch should have good mechanical and anti‐swelling properties to withstand the maximum abdominal pressure of 0.02 MPa^[^ ^31^ ^]^ during the repair of abdominal wall defect.^[^ ^32^, ^33^ ^]^ The bPVA/SIS^+^‐NP patch has good flexibility to adapt to abdominal wall movements (**Figure** **3**a–c). With a tensile strength of 0.97 MPa and a cyclic loading tensile stress of 0.23–0.29 MPa under the tensile strain of 30%, our bPVA/SIS^+^‐NP patch can meet the requirements for mechanical support in abdominal wall repair (Figure 3d,e). It is noteworthy that our bPVA/SIS^+^‐NP patch can endure a high burst pressure up to 759 mm Hg, which is greatly higher than other reported anti‐adhesion patches (Figure S7, Supporting Information).^[^ ^19^, ^26^, ^34^, ^35^, ^36^, ^37^, ^38^ ^]^ More importantly, the high burst pressure can be retained after 72 h immersion in a solution containing 0.2 mg mL^−1^ of collagenase I. In sharp contrast, the burst pressure of SIS diminishes from 448 to 29 mm Hg after 12 h immersion in the collagenase I solution, and finally cannot maintain the intact shape after 24 h due to its rapid degradation (Figure 3f). As shown in Figure 3g, ascribed to its high crosslinking density of hydrogen bonds, only a 6.95% swelling ratio is observed for our bPVA/SIS^+^‐NP patch after 7 days of immersion in PBS. In addition, SIS^+^‐NP and bPVA/SIS^+^‐NP do not show any significant deformation after 14 days of immersion in the collagenase I solution, while SIS degrades completely in collagenase I within only 1 day (Figure 3h). As shown in Figure 3i, the weight retention rates of SIS^+^‐NP and bPVA/SIS^+^‐NP are 94.7% and 95.8% after 3 days of immersion in the collagenase I solution, respectively, while SIS decreased to 0. The rapid degradation of SIS is similar to other biodegradable materials (e.g., polyhydroxyalkanoates; Figure S8, Supporting Information). These long‐term and robust mechanical performances are attributed to the excellent mechanical performance of bPVA hydrogel layer and the anti‐degradation capability of the functional coating of SIS. These above results demonstrate that bPVA/SIS^+^‐NP patch with excellent mechanical, anti‐swelling, and anti‐degradation properties in vitro might meet the mechanical requirements of long‐term abdominal implantation in vivo.

**Figure 4:**
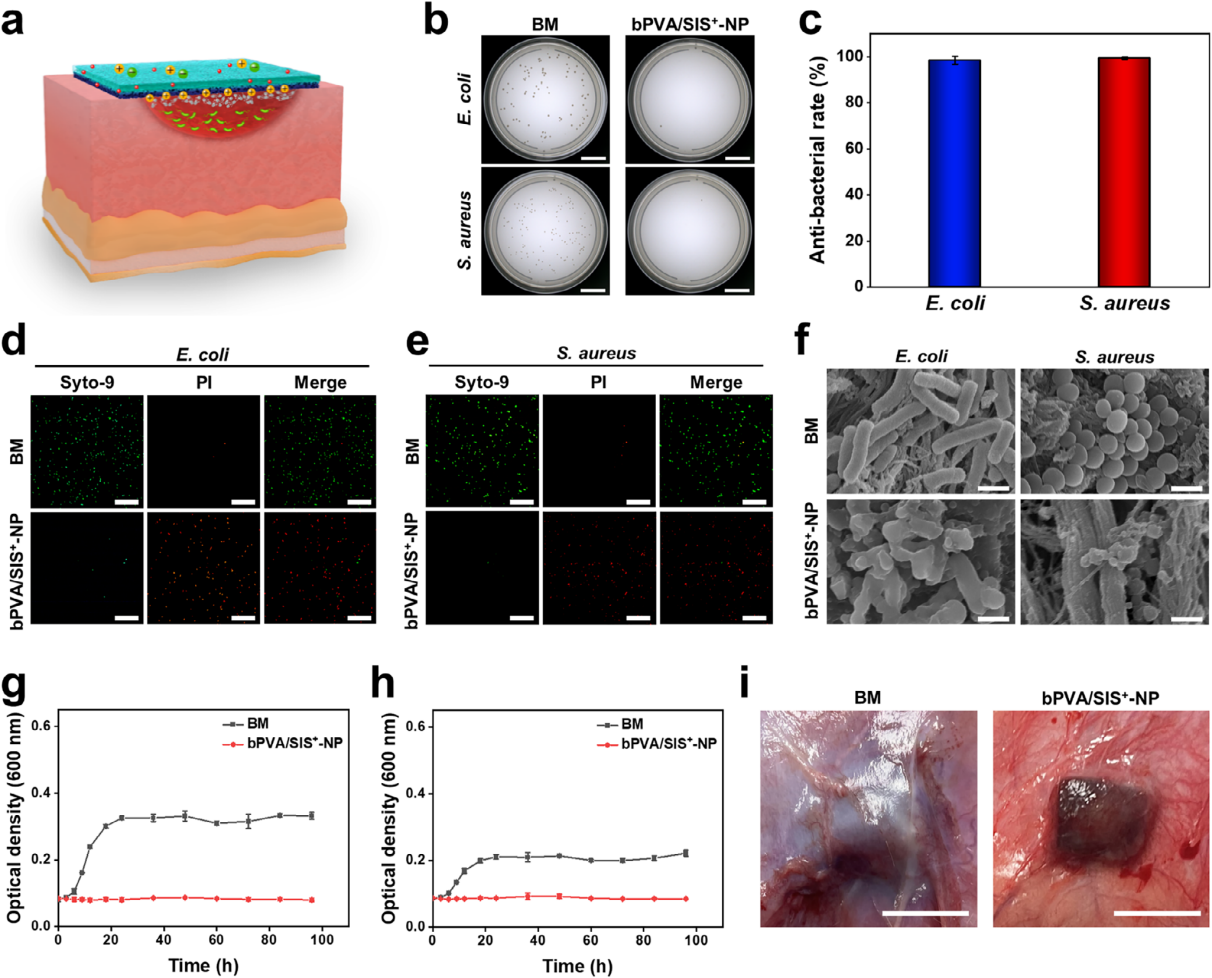
Anti‐bacterial performance of bPVA/SIS^+^‐NP patch. a) Illustration of anti‐bacterial function of the SIS^+^‐NP layer in bPVA/SIS^+^‐NP. b) Digital photos of the bacterial colonies of *E. coli* and *S. aureus* contacted with BM and bPVA/SIS^+^‐NP patches. c) The anti‐bacterial rates of BM and bPVA/SIS^+^‐NP patches against *E. coli* and *S. aureus*. d,e) Fluorescent images exhibiting the live/dead distribution of *E. coli* (d) and *S. aureus* (e) on the surfaces of BM and SIS^+^‐NP layer of bPVA/SIS^+^‐NP patches (green for live bacteria, red for dead bacteria). f) SEM images of *E. coli* and *S. aureus* after contacting with BM and bPVA/SIS^+^‐NP patches. g,h) Growth curves of *E. coli* (g) and *S. aureus* (h) as a function of culture time in the BM and bPVA/SIS^+^‐NP groups. i) Digital photos of subcutaneous infectious wounds on the back of rats treated with BM and bPVA/SIS^+^‐NP patches for 6 days. Scale bars: 2 cm in (b), 50 µm in (d, e), 1 µm in (f), and 1 cm in (i). The data are the mean ± SD (*n* = 3).

Anti‐bacterial property is the first requirement for the application of patch materials in infectious abdominal wall defect. It is well known that cationic polymer can adsorb negatively charged bacteria through electrostatic force and destroy the bacterial wall.^[^ ^39^, ^40^ ^]^ With introduction of PEI into the SIS^+^‐NP layer, the zeta potential of bPVA/SIS^+^‐NP patch can reach 23.2 mV (Figure 2k). To evaluate the anti‐bacterial performance of bPVA/SIS^+^‐NP patch, contact killing assay is conducted by adding *S. aureus* and *E. coli* culture media onto the SIS^+^‐NP layer of bPVA/SIS^+^‐NP patch. Attributing to the positive charge of PEI (**Figure** **4**a), there are almost no *S. aureus* and *E. coli* in the bPVA/SIS^+^‐NP groups, while a large number of *S. aureus* and *E. coli* are found in the commercial BM group (Figure 4b). The anti‐bacterial rates of bPVA/SIS^+^‐NP patch against *E. coli* and *S. aureus* are 98.5% and 99.4%, respectively (Figure 4c). Fluorescence staining images show that *E. coli* and *S. aureus* adhering to SIS^+^‐NP layer of bPVA/SIS^+^‐NP patch are essentially dead (Figure 4d,e). SEM images further exhibit that the morphologies of *E. coli* and *S. aureus* in the bPVA/SIS^+^‐NP group is extraordinarily distorted and incomplete, while those in the BM group can maintain their intact cellular morphologies with a smooth surface (Figure 4f). As shown in Figure 4g,h, the optical density (OD_600_) values of both *S. aureus* and *E. coli* in the commercial BM group significantly increase from 9 h, and reach their peak at 24 h. In contrast, the OD_600_ values of *S. aureus* and *E. coli* in the bPVA/SIS^+^‐NP group do not increase during the measurement time of 96 h, indicating that almost all *S. aureus* and *E. coli* are killed. Furthermore, after treating subcutaneous infectious wounds with different patches on the back of rats for 6 days, we found that there is no abscess formation in the bPVA/SIS^+^‐NP group, while obvious abscess fluid accumulation can be observed in the commercial BM group (Figure 4i). The above results show that the introduction of polyethyleneimine endows bPVA/SIS^+^‐NP patch with strong positive charge to achieve excellent anti‐bacterial property.

**Figure 5:**
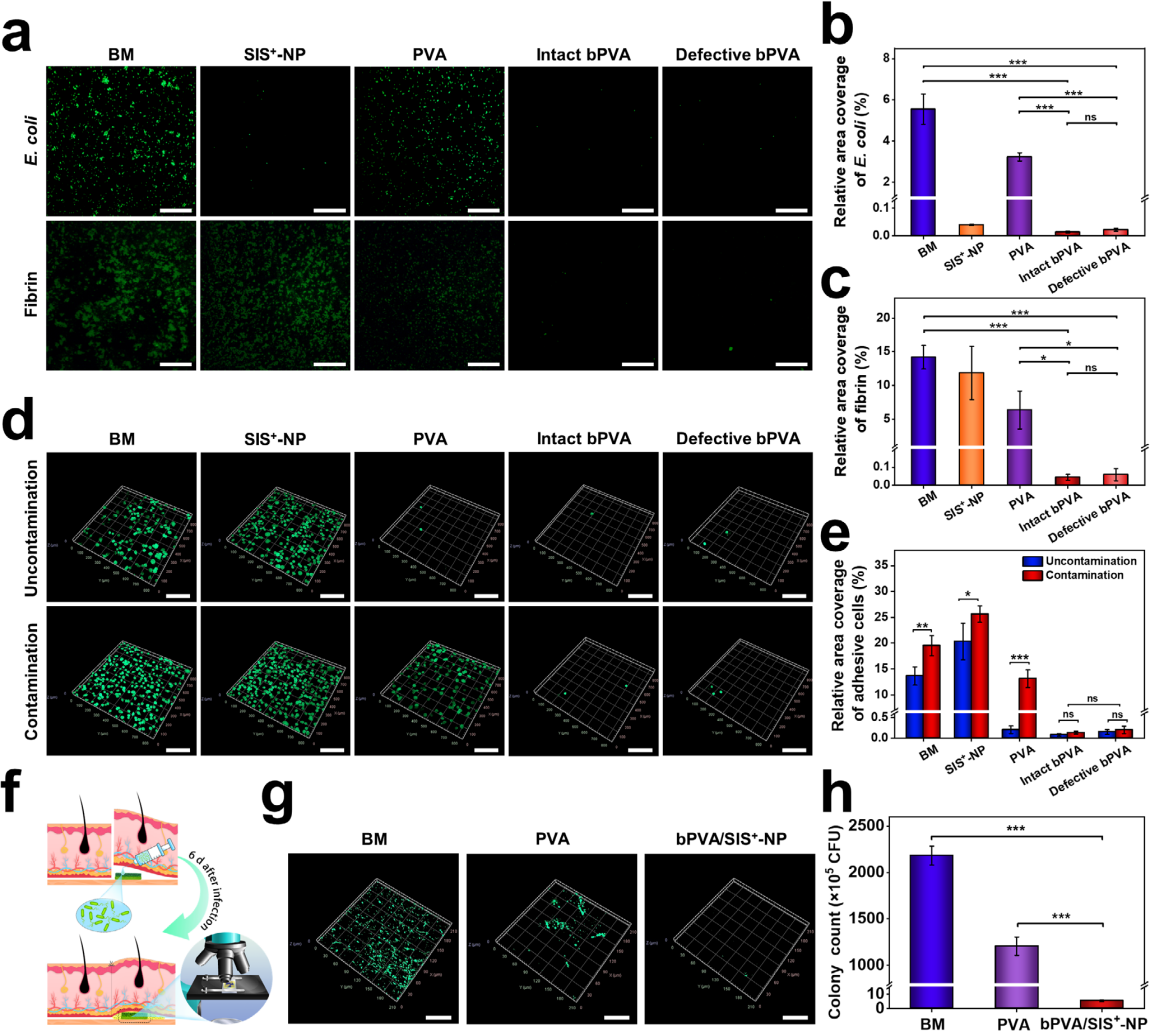
The anti‐contamination property of bPVA/SIS^+^‐NP patch. a–c) Comparison of anti‐contamination performance against *E. coli* (a, b) and fibrin (a, c) for commercial BM, PVA hydrogel, as well as SIS^+^‐NP, intact bPVA, and defective bPVA layers of bPVA/SIS^+^‐NP patch. d,e) Comparison of anti‐adhesion performance toward L929 cells for commercial BM, PVA hydrogel, as well as SIS^+^‐NP, intact bPVA, and defective bPVA layers of bPVA/SIS^+^‐NP patch with or without contamination of dead *E. coli* and fibrin. f) Illustration of commercial different samples implanted in subcutaneous infectious wounds on the back of rats for 6 days. g,h) 3D fluorescent images (g) and corresponding statistical data (h) of bacteria on the surfaces of commercial BM, PVA hydrogel, and bPVA/SIS^+^‐NP patch implanted in subcutaneous infectious wounds on the back of rats for 6 days. Scale bars: 100 µm in (a), 200 µm in (d), and 50 µm in (g). The data are the mean ± SD (*n* = 3; * *p* < 0.05, ** *p* < 0.01, *** *p* < 0.001).

The contamination of bacteria and deposited proteins for repair material can cause severe visceral adhesion and delay defect healing, so the stable anti‐contamination properties of repair materials are crucial for their application in infectious microenvironment.^[^ ^41^, ^42^, ^43^ ^]^ Conventional anti‐contamination strategies often employ surface coating and copolymerization approaches. For example, commercial Parietex composite (PCO) patch is composed of a polyester‐based mesh and an outer collagen film. The coating is prone to fall off due to the poor interfacial interactions. To evaluate the stable anti‐contamination property of our bPVA/SIS^+^‐NP patch, green fluorescent protein (GFP)‐expressing *E. coli and* 6‐FAM SE‐labeled fibrin are selected as representative contamination sources to co‐incubate with different samples. As shown in **Figure** **5**a–c, compared with commercial BM (≈5.55% surface coverage *for E. coli and* ≈14.18% surface coverage for fibrin) and PVA hydrogel patch (≈3.23% surface coverage for *E. coli* and ≈6.36% surface coverage for fibrin), intact bPVA layer of bPVA/SIS^+^‐NP patch exhibits significantly less contamination of *E. coli* (≈0.01% surface coverage) and fibrin (≈0.04%). More importantly, after scraping part of bPVA layer of bPVA/SIS^+^‐NP patch with scalpel (Figure S9, Supporting Information), the defective bPVA layer of bPVA/SIS^+^‐NP patch can still maintain excellent anti‐contamination against *E. coli* (≈0.02% surface coverage) and fibrin (≈0.06% surface coverage). To further elucidate the anti‐adhesion performance of our bPVA/SIS^+^‐NP patch in the infectious environment, after immersing samples into a bio‐contaminated solution containing heat‐inactivated bacteria and fibrin for 12 h, L929 cells that play the key role in visceral adhesion are seeded onto the surface of different samples for 12 h. As shown in Figure 5d,e, with and without contamination of heat‐inactivated *E. coli* and fibrin, commercial BM and SIS^+^‐NP can cause a large number of L929 cell adhesion. In contrast, few L929 cells can be found on the intact (≈0.08% surface coverage for uncontamination and ≈0.13% surface coverage for contamination) and defective (≈0.15% surface coverage for uncontamination and ≈0.20% surface coverage for contamination) bPVA layers of our bPVA/SIS^+^‐NP patch with and without contamination by heat‐inactivated *E. coli* and fibrin. It is worth mentioning that PVA hydrogel can cause noticeable L929 cell adhesion (≈13.10% surface coverage) when contaminated by the dead *E. coli* or fibrin, although it can prevent L929 cell adhesion (≈0.20% surface coverage) without contamination. Moreover, to verify the in vivo anti‐contamination performance of bPVA/SIS^+^‐NP patch, we further examined the bio‐film and bacterial adhesion on the surface of various patches after implantation in subcutaneous infectious wounds on the back of rats for 6 days (Figure 5f). There is almost no bacterial adhesion and bio‐film formation on the bPVA layer of bPVA/SIS^+^‐NP patch, while obvious bacterial adhesion and bio‐film formation could be observed on the surfaces of the commercial BM and PVA hydrogel (Figure 5g,h; Figure S10, Supporting Information). These above results indicate that the strong hydrogen bonding network of bPVA layer with PSBMA zwitterionic side chains and its strong interfacial binding force with SIS^+^‐NP layer can achieve stable anti‐contamination property and the corresponding visceral anti‐adhesion performance in the infectious environment.

**Figure 6:**
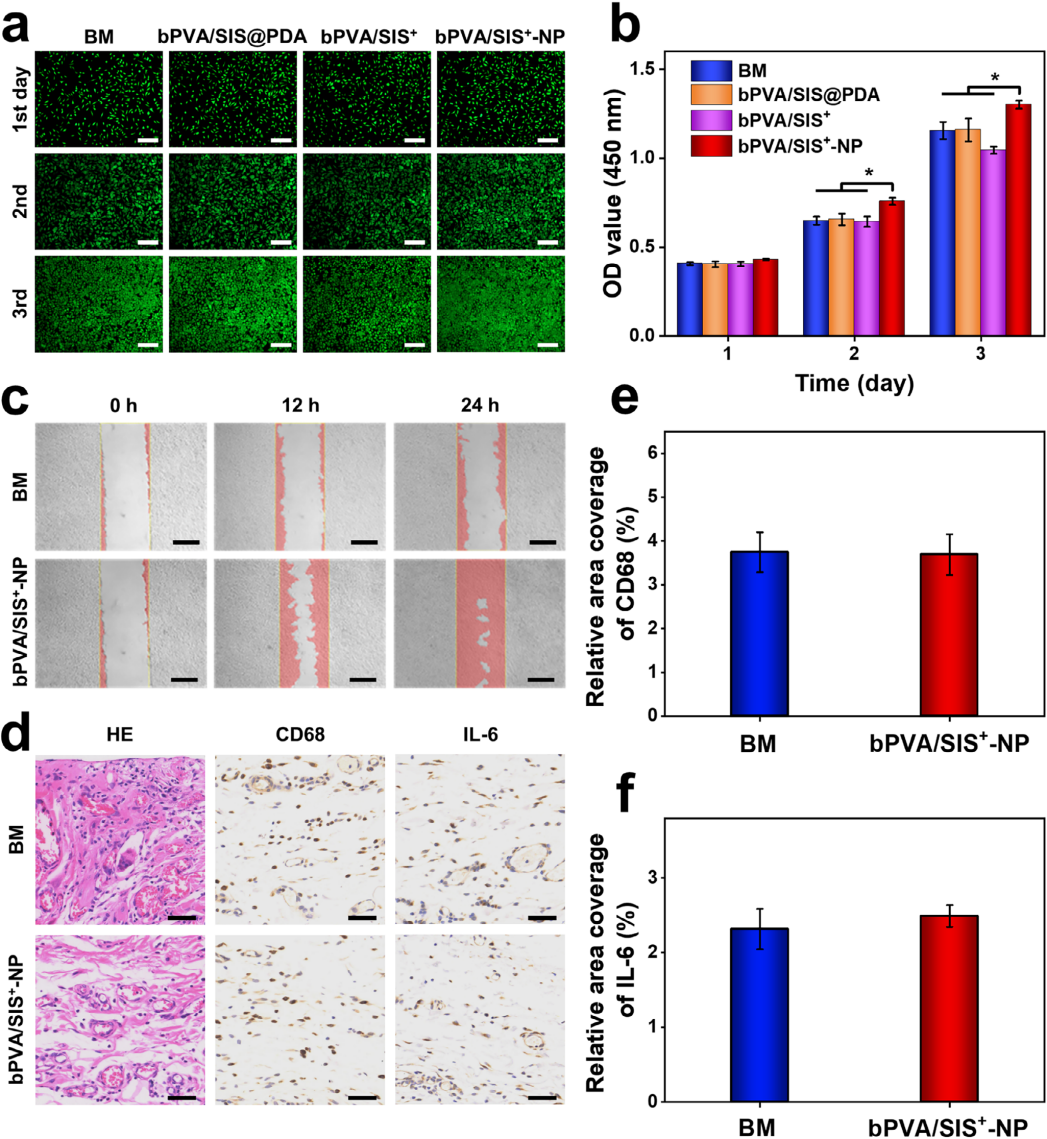
Biocompatibility and cell migration promoting properties of bPVA/SIS^+^‐NP patch. a,b) In vitro fluorescence images (a) and CCK‐8 (b) of L929 cells cultured in the extract solutions of BM, bPVA/SIS@PDA, bPVA/SIS^+^, and bPVA/SIS^+^‐NP patches for 1, 2, and 3 days. c) Images of scratch test of L929 cells treated with the extract solutions of BM and bPVA/SIS^+^‐NP patches. d) HE staining and immunohistochemical staining of CD68 and IL‐6 after subcutaneous tissue implantation for 5 days. e,f) Quantitative analysis of CD68 (e) and IL‐6 (f). Scale bars: 200 µm in (a), 500 µm in (c), and 50 µm in (d). The data are the mean ± SD (*n* = 3; * *p* < 0.05).

Excellent biocompatibility is of great significance for the repair materials of infectious abdominal wall defect. But there is an inherent trade‐off between anti‐microbial activity and cell proliferation.^[^ ^23^, ^44^ ^]^ In vitro biocompatibility is evaluated using cell proliferation assay and live/dead staining assay. Compared to the bPVA/SIS@PDA group, the bPVA/SIS^+^ group with anti‐bacterial property exhibits less L929 cells on the 1st, 2nd, and 3rd days, indicating that the addition of PEI can reduce the proliferation of cells. In order to improve the pro‐healing ability, as a key coenzyme of energy metabolism for cell proliferation and migration,^[^ ^30^, ^45^, ^46^ ^]^ NP is introduced into bPVA/SIS^+^‐NP patch. Due to electrostatic interaction, bPVA/SIS^+^‐NP patch can slowly release NP (Figure S11, Supporting Information), and the number of L929 cells in the bPVA/SIS^+^‐NP group is much higher than the bPVA/SIS^+^ group on the 2nd and 3rd days (**Figure** **6**a,b). Rabbit red blood cells are utilized to assess the hemolytic activity of bPVA/SIS^+^‐NP patch. The positive control group where red blood cells mix with deionized water shows complete hemolysis and continued red color after centrifugation (Figure S12, Supporting Information). By contrast, the negative control group where red blood cells mix with PBS solution, and the bPVA/SIS^+^‐NP groups do not show any hemolysis with a clear separation of red blood cells after centrifugation. The hemolysis rate of the bPVA/SIS^+^‐NP group is determined to be 3.09%, which falls within the standard range specified for hemolytic tests (< 5%). Moreover, compared with the BM group, cells in the bPVA/SIS^+^‐NP group show significant convergence at 12 and 24 h, indicating that the encapsulated NP also contributes to cell migration (Figure 6c). To further evaluate the compatibility of bPVA/SIS^+^‐NP patch in vivo, we have implanted BM and bPVA/SIS^+^‐NP patches into the subcutaneous tissue of a rat model for 5 days, and performed histological analysis for local inflammatory response. Hematoxylin and eosin (HE) staining results show that the BM and bPVA/SIS^+^‐NP groups do not cause obvious inflammatory cell infiltration (Figure 6d). Immunohistochemical staining results show no significant difference in the expression levels of CD68 and IL‐6 between the BM and bPVA/SIS^+^‐NP groups (Figure 6e,f). The above results suggest that the introduction of NP endows our anti‐bacterial bPVA/SIS^+^‐NP patch with excellent biocompatibility and cell migration promoting properties.

**Figure 7:**
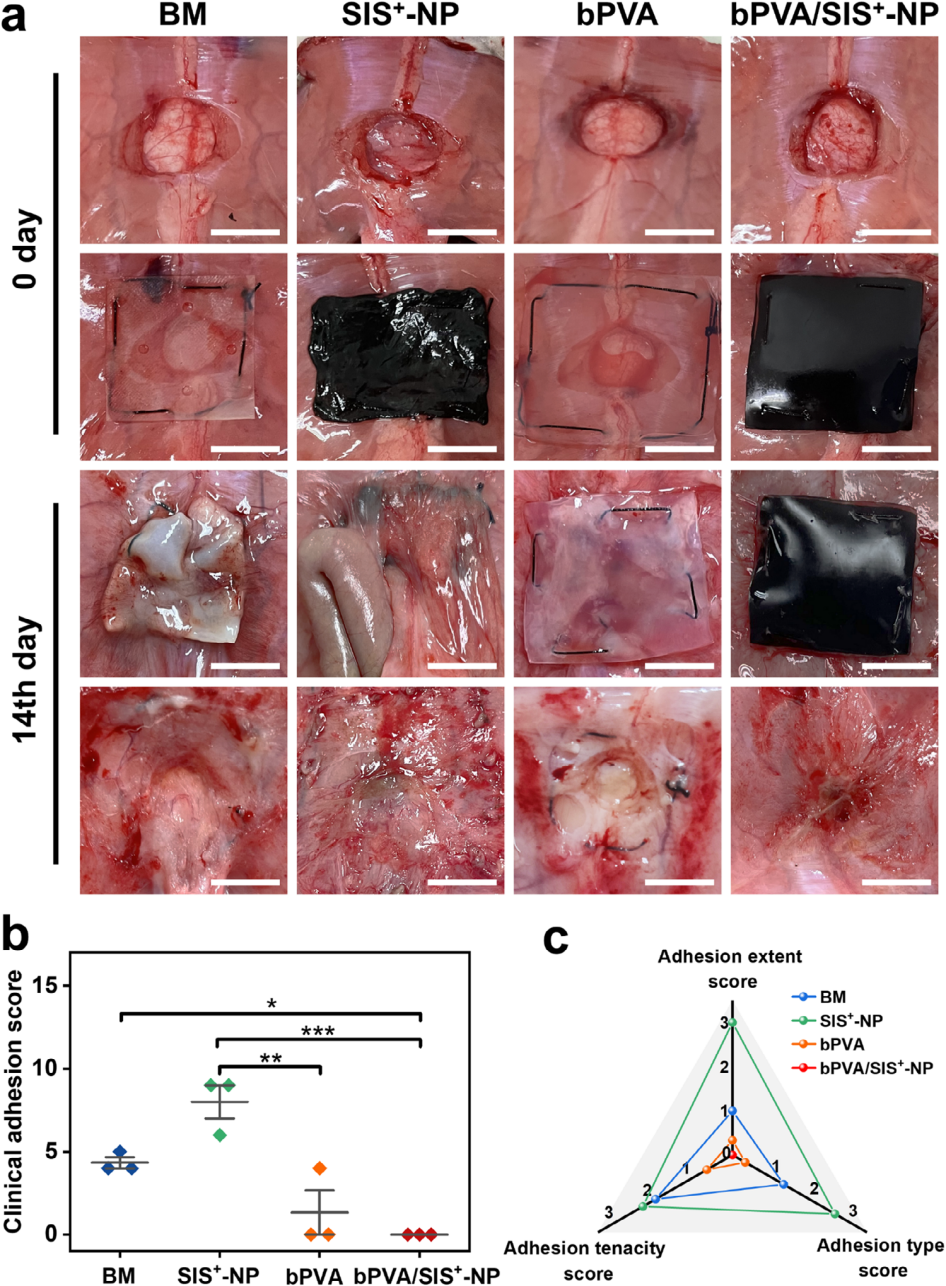
The anti‐adhesion property of bPVA/SIS^+^‐NP patch in vivo. a) Digital photos of visceral adhesion formation and wound healing on the 14th day of repairing contaminated abdominal wall defect with the BM, SIS^+^‐NP, bPVA, and bPVA/SIS^+^‐NP patches. b) Quantitative scoring analysis of overall visceral adhesions in abdominal wall defect treated with different patches on the 14th day after surgery. c) Quantitative scoring analysis of adhesion extent, adhesion type, and adhesion tenacity of abdominal wall defect treated with different patches on the 14th day after surgery. Scale bars: 1 cm in (a). The data are the mean ± SD (*n* = 3; * *p* < 0.05, ** *p* < 0.01, *** *p* < 0.001).

To evaluate visceral anti‐adhesion and pro‐healing properties of bPVA/SIS^+^‐NP patch in vivo, 10 mm‐diameter full‐thickness abdominal wall defect is constructed on rats and then infected with 100 µL *E. coli* fluid to construct the contaminated abdominal wall defect models (Figure S13, Supporting Information). Then the abdominal wall defect is repaired with different patches, including BM, bPVA, SIS^+^‐NP, and bPVA/SIS^+^‐NP patches. For bPVA/SIS^+^‐NP patch with double layers, the SIS^+^‐NP layer is toward the abdominal wall defect. On the 14th day after surgery, the rats were humanely euthanized and visceral adhesion was assessed by clinical grades, including degree of adhesion, type of adhesion, and strength of adhesion (Table S1). Digital photos of visceral adhesions and wound healing in different treatment groups are shown in **Figure** **7**a and corresponding quantitative scoring is recorded in Figure 7b,c. The SIS^+^‐NP group with loose porous structure and cationic anti‐bacterial property cannot effectively prevent bio‐contamination, unavoidably exhibiting moderate visceral adhesion, although it can control local infection and promote wound healing. In contrast, due to the synergistic effect of its dense structure and grafted PSBMA zwitterionic side chains, the bPVA group does not show any visceral adhesion, but suffers from poor infection control and wound healing. To find a balance between anti‐bacterial and anti‐adhesive properties, bPVA/SIS^+^‐NP patch is composed of bPVA layer and SIS^+^‐NP layer. The bPVA/SIS^+^‐NP group not only shows no significant visceral adhesions, but also can kill bacteria and promote wound healing (Figure 7b,c).

**Figure 8:**
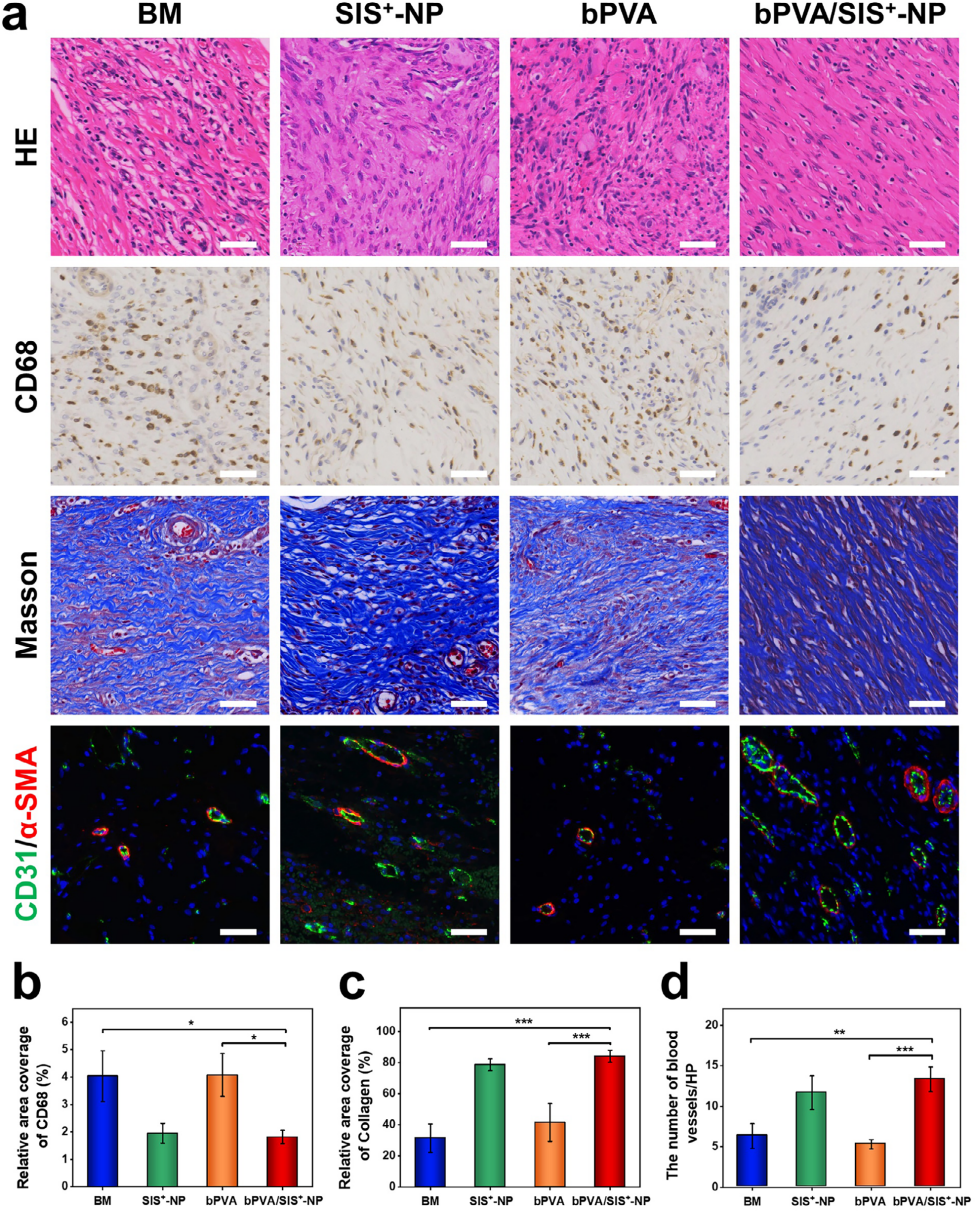
Pro‐healing performance of bPVA/SIS^+^‐NP patch in rat infectious abdominal wall defect model. a) HE staining and immunohistochemical images of CD68 staining, Masson staining, and CD31/α‐SMA for the BM, SIS^+^‐NP, bPVA, and bPVA/SIS^+^‐NP patches. Scale bars: 50 µm. b–d) Quantitative analysis of CD68 (b), collagen (c), and wound blood vessels (d). The data are the mean ± SD (*n* = 3; * *p* < 0.05, ** *p* < 0.01, *** *p* < 0.001).

Histological analysis is further conducted to evaluate the pro‐healing property of bPVA/SIS^+^‐NP patch. As shown in **Figure** **8**a, HE staining and immunohistochemical staining images reveal that both the SIS^+^‐NP and bPVA/SIS^+^‐NP groups exhibit a significant reduction in inflammatory cell infiltration and a notable decrease in CD68 expression compared to the BM and bPVA groups, attributed to the good biocompatibility of SIS^+^‐NP and the effective anti‐infection properties of its polycationic PEI components (Figure 8b). As one of the most important components of extracellular matrix, collagen is an important indicator for evaluating tissue healing. And angiogenesis is crucial in wound repair and can be studied through dual immunofluorescence staining of CD31 (endothelial cell marker) and α‐SMA (alpha smooth muscle actin marker). With slow release of NP, the bPVA/ SIS^+^‐NP and SIS^+^‐NP groups exhibit much more collagen deposition and higher expression of CD31 and α‐SMA than those of the BM and bPVA groups (Figure 8c,d). Therefore, our bPVA/SIS^+^‐NP patch can integrate excellent anti‐infection, anti‐adhesion, and pro‐healing properties, which are valuable for efficient repair of infectious abdominal wall defect.

## 3 Conclusion

In this study, we have successfully developed a zwitterionic polymer brush‐based asymmetric biological patch through a self‐induced phase separation based united strategy. Coated with drug loaded PDA/PEI, the cationic SIS^+^‐NP layer not only shows high anti‐bacterial rates against *E. coli* and *S. aureus*, but also enables prolonged release of a pro‐healing factor by electrostatic interaction. The bPVA hydrogel layer with the PSBMA zwitterionic side chains not only can prevent contamination and visceral adhesion, but also can form strong mechanical interlocking with SIS^+^‐NP layer to achieve a high and stable burst pressure tolerance. With such merits, our multifunctional biological patch can achieve comprehensive control of the complex microenvironment of infectious abdominal wall defect. We hope that this work can provide a new direction in biofriendly preparation of high‐performance biological patches for effective repair of infectious soft‐tissue defects.

## 4 Experimental Section

The Experimental Section is available in the Supporting Information.

## Conflict of Interest

The authors declare no conflict of interest.

## Supporting information



Supporting Information

## Data Availability

The data that support the findings of this study are available from the corresponding author upon reasonable request.
