# Supplementary material for: Polymer Brush‐Based Asymmetric Porous Patch with Excellent Anti‐Bacterial and Anti‐Contamination Properties Enables Infectious Abdominal Wall Defect Repair
Source: Adv Sci (Weinh). 2025 Apr 7;12(25):2500865. doi: 10.1002/advs.202500865 (PMC12224944; doi:10.1002/advs.202500865)
Supplement: Supplementary file 1 — Supporting Information [file ADVS-12-2500865-s001.docx]

### Supporting Information

**Polymer Brush-Based Asymmetric Porous Patch with Excellent Anti-Bacterial and Anti-Contamination Properties Enables Infectious Abdominal Wall Defect Repair**

Zeping Huang^a,1^, Weiwen Liang^a,1^, Binying Peng^a,1^, Yang Ouyang^a,1^, Zixin Chen^a^, Mawsoom Mamut^b^, Zhipeng Huang^b^, Hui Wang^a^, Rongkang Huang^a,^*, Bo Ma^c,^*, Bingna Zheng^d,^*, Jian Cai^e,^*

^a^ Department of General Surgery (Colorectal Surgery), Guangdong Institute of Gastroenterology, Biomedical Innovation Center, Guangdong Provincial Key Laboratory of Colorectal and Pelvic Floor Diseases, The Sixth Affiliated Hospital, Sun Yat-sen University, Guangzhou 510655, P. R. China

^b^ Department of Urology, The First People's Hospital of Kashi, Kashi 844099, P. R. China

^c^ Department of Urology, The Sixth Affiliated Hospital, Sun Yat-sen University, Guangzhou 510655, P. R. China

^d^ The Eighth Affiliated Hospital, Sun Yat-sen University, Shenzhen 518000, P. R. China

^e^ Department of Colorectal Surgery, Shenzhen Second People’s Hospital, First Affiliated Hospital of Shenzhen University, Medical Innovation Technology Transformation Center of Shenzhen Second People's Hospital, Shenzhen University, Shenzhen 518035, P. R. China

* Corresponding authors: Rongkang Huang, Bo Ma, Bingna Zheng, Jian Cai

E-mails:

[huangrk3@mail.sysu.edu.cn](mailto:huangrk3@mail.sysu.edu.cn) (Rongkang Huang)

[mabo3@mail.sysu.edu.cn](mailto:mabo3@mail.sysu.edu.cn) (Bo Ma)

[zhengbn3@mail.sysu.edu.cn](mailto:zhengbn3@mail.sysu.edu.cn) (Bingna Zheng)

[caijian_dr@email.szu.edu.cn](mailto:caijian_dr@email.szu.edu.cn) (Jian Cai)

^1^ These authors contributed equally: Zeping Huang, Weiwen Liang, Binying Peng, Yang Ouyang

**Experimental Section**

***Materials***

Polyvinyl alcohol 1799 (PVA-1799, degree of alcoholysis 98~99 mol%, water content ~1.4 wt%), dimethyl sulfoxide (DMSO), dopamine (DA), ethylene diamine tetraacetic acid (EDTA), sodium hydroxide (NaOH), hexafluoroisopropanol, and sodium chloride (NaCl) were purchased from Shanghai Macklin Biochemical Technology Co., Ltd. (China). Hydrogen chloride (HCl) was purchased from Guangzhou Chemical Reagent Factory (China). Sulfobetaine methacrylate (SBMA) was purchased from Shanghai Yuanye Bio-Technology Co., Ltd. (China). Polyhydroxyalkanoates (PHA) was purchased from MedPHA Bioscience Co., Ltd. (China). Ceric ammonium nitrate (CAN) and polyethyleneimine (PEI) were purchased from Shanghai Aladdin Biochemical Technology Co., Ltd. (China). Collagenase I was purchased from neoFroxx Co., Ltd. (GER). The Cell Counting Kit-8 (CCK-8), phosphate buffer saline (PBS, 0.01 M, pH 7.4), and Calcein AM/PI Double Staining Kit were purchased from Dalian Meilun Biotech Co., Ltd. (China). L929 cells were provided by the Procell Life Science & Technology Co., Ltd. (China). Male Sprague-Dawley rats (250~300 g in weight) were provided by laboratory animal center of Sun Yat-sen University (China). Fetal bovine serum (FBS) and dulbecco’s modified eagle medium (DMEM) were purchased from Gibco-BRL Life Technologies, Inc. (USA). Carboxyfluorescein N-hydroxysuccinimide ester (6 FAM SE) was purchased from ApexBio Technology LLC (USA). Nicotinamide phosphoribosyltransferase (NP) was provided by Beijing Solarbio Science & Technology Co., Ltd. (China). NP ELISA kit was provided by Jiangsu Kewide Biotech Co., Ltd. (China). Live/Dead Bacterial Double Stain Kit was purchased from Shanghai Fushen Biotechnology Co., Ltd. (China). All solvents and chemicals were purchased from commercial sources and used without further purification.

***Preparation of SIS patch***

The small intestinal submucosa (SIS) was obtained via established protocols^[1]^. The fresh small intestine of a pig procured from the commercial market was extensively washed with water to remove its contents. Subsequently, the mucosal layer, serosal layer, and muscular layer of the small intestine were physically removed. For decellularization treatment, the tissue was sequentially immersed in a solution containing 100 mM ethylene diamine tetra acetic acid (EDTA) and 200 mM NaOH for 16 h, a solution containing 1 M NaCl and 1 M HCl for 8 h, and a PBS solution containing 1 M NaCl for 16 h. Subsequently, it was immersed in 5% peracetic acid for 4 h and incubated in PBS for 2 h. After rinsing with deionized water, the tissue was immersed in 20% ethanol containing 0.1% peracetic acid for 8 h and then in deionized water for 2 h. The SIS patch was stored at 4°C.

***Preparation of*** ***SIS^+^ patch***

Dopamine hydrochloride was dissolved in Tris buffer, and then SIS was immersed in dopamine solution with gentle stirring for 24 h. The as-obtained sample was extensively washed with water under ultrasonication, and subsequently soaked in a 4 g L^‑1^ PEI aqueous solution for 6 h. SIS^+^ was obtained after gently rinsing with water three times to remove unbound PEI, and stored in dark for further use.

***Synthesis of PVA-g-PSBMA polymer brush (bPVA)***

PVA (10 g) was dissolved in DMSO (100 mL) at 60°C. Ceric ammonium nitrate (CAN, 0.25 g), served as the initiator, was dissolved in DMSO (5 mL). Then, SBMA monomers (5, 10, and 15 g) were added to the PVA solution, and the CAN solution was slowly added during the 30 min of nitrogen bubbling. After polymerization at 60°C for 19 h, the reaction was completed and the solution was cooled down to 25°C. The polymer was precipitated with anhydrous ethanol and washed daily with deionized water for 3 days to remove residual ions. The bPVA was then dried for future applications. With the different weight ratios of PVA and SBMA (2:1, 2:2, and 2:3), the as-synthesized bPVAs were named as bPVA1, bPVA2, and bPVA3, respectively. Unless otherwise specified, the bPVA used in this paper is bPVA2.

***Preparation of bPVA/SIS^+^-NP patch***

20 g of bPVA2 was dissolved in 80 mL of DMSO by heating and stirring for 3 h at 60°C in an oil bath. After complete dissolution in DMSO and cooling to 25°C, the solution was cast into a mold, and the SIS^+^ patch was spread on the upper layer of the PVA solution. A tightly bonded bilayer asymmetric hydrogel (bPVA/SIS^+^) was obtained by standing at 24°C for 12 h, drying in an air-drying oven at 37°C for 24 h, and soaking in deionized water at 24°C for 48 h. The water was changed every 8 h to thoroughly remove DMSO. The bPVA/SIS^+^ patch was trimmed to a square of 2 cm × 2 cm. 100 µL of NP suspension (10 μg mL^‑1^) was dropped onto the porous SIS^+^ surface in a 12-well plate, and the patch was stored at 4°C for 24 h to eventually produce the bPVA/SIS^+^‑NP patch.

***Preparation and degradation of PHA patch***

PHA was dissolved in hexafluoroisopropanol to form a 10wt% solution. The electrospinning was carried out with an injection rate of 1.5 mL h^-1^, a voltage of 24 kV, a distance of 15 cm between the needle and the drum, a drum speed of 1000 rpm, a temperature of 25℃, and a duration of 3 h. PHA patch was obtained after removing solvents in a vacuum dryer. Degradation experiment was conducted in 0.2 mg mL^-1^ lipase, and the remaining mass of the patch was measured at regular intervals.

***Characterization***

The chemical structures were confirmed by Fourier transform infrared spectroscopy with attenuated total reflectance (ATR-FTIR) (Spectrum 100, Perkin Elmer, USA), and 400 MHz ^1^H NMR spectrometer (AVANCE III HD 400, Bruker, Germany) using D_2_O as the solvent for analysis. The hydrophilicity of the material was studied using a contact angle meter (Attension Theta Lite 100, Biolin Scientific). The microstructure of hydrogels was analyzed using field emission scanning electron microscopy (FE-SEM, S-4800, Hitachi). Sample preparation involved rapid freezing of the specimens in liquid nitrogen, lyophilization, and platinum coating before microscopic analysis. Morphological characterization of hydrated samples was conducted using an Olympus DSX-1000 digital optical microscope. The zeta potential of samples was determined using a Zetasizer Nano-ZS PN3702 system (Malvern Instruments, Worcestershire, England) at 25°C. Prior to analysis, the samples were lyophilized, pulverized, and then dispersed in deionized water for 10 min. The dispersions were filtered to eliminate any oversized hydrogel fragments before zeta potential measurement.

***Mechanical performances***

*Tensile test*

All patches were cut into rectangular strips. Tensile tests were performed on a universal mechanical testing machine (WD-5A, Guangzhou Experimental Instrument Factory, China) at the stretching velocity of 50 mm min^‑1^. The cyclic tensile test involved loading at a uniform rate of 30 mm min^‑1^ to a strain of 30% for 30 cycles.

*Burst pressure test*

Patches were fixed in a mold with a window, of which the diameter was 20 mm. The mold was connected to a 60 mL syringe filled with water. The burst pressure was measured by pumping deionized water with the syringe at a rate of 5 mL min^‑1^ and the maximum pressure (burst pressure) was recorded with a digital pressure gauge (HT-1895, Xintest, China). To investigate the changes in burst pressure across the degradation period, bPVA/SIS^+^-NP patch (20 mm × 20 mm) was immersed in a PBS media supplemented with 0.2 mg mL^‑1^ collagenase I and subjected to agitation on shaker (37 ℃ and 200 rpm) to initiate the degradation experiment. The burst pressure at different time points was recorded.

*Bonding strength test*

For the testing sample of bPVA/SIS^+^-NP patch, the intermediate interconnection area between SIS^+^-NP and bPVA layers was 1 cm × 1 cm, and the two ends were composed of a single layer (SIS^+^-NP or bPVA layer) without interconnections. The end of bPVA layer was fixed, while a 200 g load was fixed at the end of SIS^+^-NP layer to evaluate the bonding strength between the two layers.

***Swelling behavior***

Swelling ratios of bPVA/SIS^+^-NP patch was assessed via a gravimetric analysis. The air-dried samples were immersed in deionized water for 24 h as a starting point for the test. Subsequently, they were transferred to PBS (pH 7.4) and maintained at 37 ℃. At specified intervals, the samples were retrieved, excess solution was removed from the surface using filter paper, and then the samples were weighed. The swelling rate was calculated according to the following formula:

$$Swelling rate\left（ \% \right）=\frac{\left（ W_{2} - W_{1} \right）}{W_{1}} \times100$$

where *W*_1_ represents the starting weight of the sample after 24 h of rehydration, while *W*_2_ represents the weight of the sample post-swelling.

## *In vitro* *anti-bacterial property*

To investigate the anti-bacterial activity of bPVA/SIS^+^-NP patch, *S. aureus* and *E. coli* were used for the tests. The bacterial solution was further diluted to 10^7^ CFU mL^‑1^, and 100 μL of the *E. coli* and *S. aureus* were added to groups of BM, bPVA/SIS^+^, and bPVA/SIS^+^-NP patches (size: 20 mm × 20 mm), cultured at 37°C with shaking (180 rpm) for 3 h. After adding 1 mL PBS to the samples and ultrasonication, the solution was diluted 10 times and then dropped on the plate. Each sample was repeated 3 times.

For fluorescent imaging, 100 μL of *S. aureus* or *E. coli* suspension (10^7^ CFU mL^‑1^) was seeded onto different samples and cultured for 3 h at 37°C. Subsequently, the samples were stained with live/dead bacterial double stain kit, and the incubation was continued at room temperature for 15 min in dark. Finally, the samples were visualized using a fluorescence microscope (Olympus IX73, Japan). For SEM imaging, after 3 h of bacterial inoculation, the sample was washed with PBS and immersed overnight in 2.5% glutaraldehyde at 4°C. Subsequently, the samples were subjected to gradient dehydration using a series of ethanol aqueous solutions (30%, 50%, 70%, 80%, 90%, and 100% v/v), and each step lasted 15 min. After sufficient freeze-drying, samples were fixed using conductive tape on the sample stage, and then sprayed with platinum. The morphology of the bacteria was observed by SEM.

To test the long-lasting anti-bacterial properties of bPVA/SIS^+^-NP patch, *S. aureus* or *E. coli* suspensions (4 mL, 10^5^ CFU mL^-1^) were added to small glass bottles containing BM and bPVA/SIS^+^-NP patches (size: 20 mm × 20 mm), respectively. In this test, the untreated bacterial solution was used as the control group, and each group was repeated three times. The bottle was incubated at 37°C at 180 rpm for 96 h. During the culture process, the optical density (OD) values at 600 nm of the above bacterial solutions were measured at different time intervals.

***In vivo anti-bacterial*** ***property***

The animal use protocol listed below has been reviewed and approved by the Institutional Animal Care and Use Committee (IACUC), Sun Yat-Sen University (China, license number: SYSU-IACUC-2024-001052). Adult male Sprague-Dawley rats (250~300 g, Sun Yat-sen University) were used for all in vivo studies.

In order to further test the anti-bacterial property of bPVA/SIS^+^-NP patch in deep abscess wounds in vivo, the BM, PVA, and bPVA/SIS^+^-NP patches were implanted into the back skin of rats and injected with 100 μL *E. coli* (10^7^ CFU mL^-1^) to contaminate the wound. After 6 days, the rats were euthanized and the patch implantation sites were photographed to evaluate the degree of wound abscess.

***Anti-biocontamination properties***

*Anti-adhesion of bacteria*

An engineered *Escherichia coli* (*E. coli*) strain that constitutively expresses green fluorescent protein (GFP) was prepared by following the previously reported protocol and cultured in Luria-Bertani broth (LB broth) overnight at 37°C. 10 µL *E. coli* suspension diluted in 1 mL of fresh LB broth, was applied to 1 cm × 1 cm substrates and incubated for 12 h at 37°C. After incubation, the samples were rinsed gently with PBS to eliminate non-adherent *E. coli* and then examined using a fluorescence microscope. The extent of *E. coli* adhesion on the substrates was quantified using Image J software.

*Anti-deposition of fibrin*

The samples (1 cm × 1 cm) were incubated with 1 mg mL^‑1^ 6-FAM SE labeled fibrin at 37°C in a shaker (220 rpm) for 12 h. The sample was gently washed in PBS and fixed in 2.5 v/v% glutaraldehyde for 1 h. Then image the sample using a fluorescence microscope and analyze it using Image J software.

*Anti-adhesion of cells*

The bPVA layer of bPVA/SIS^+^-NP patch surface was gently scraped off by a blade to prepare the defective bPVA layer. The samples (including commercial BM, PVA hydrogel, as well as SIS^+^-NP, intact bPVA, and defective bPVA layers of bPVA/SIS^+^-NP patch) were cut into the same size of the well of a 48-well plate. For the uncontamination group, the samples were incubated with PBS solution (10 μM) at 37℃ for 12 h. For the contamination group, the samples were incubated with a mixed contaminated solution containing *E. coli* inactivated by high-pressure steam (10^7^ CFU mL^-1^) and fibrin (1 mg mL^-1^). L929 cells were seeded on the surfaces of commercial BM, PVA hydrogel, as well as SIS^+^-NP, intact bPVA, and defective bPVA layers of bPVA/SIS^+^-NP patch at a density of 3 × 10^4^ cells per well. Calcein-AM kit was used for staining cells on the samples for 20 min at 37°C. The samples were then imaged with a fluorescence microscope and analyzed by using Image J software.

***In vitro biocompatibility***

The samples were immersed in tubes filled with 3 mL of culture medium and subjected to incubation at 37°C for 24 h. The subsequent in vitro biocompatibility analysis involved the evaluation of cytocompatibility and cell proliferation.

Cytocompatibility was determined utilizing a live/dead staining protocol, and cell proliferation was assessed via the CCK-8 assay. L929 cells were inoculated onto 96-well plates at a seeding density of 3 × 10^3^ cells per well, each containing 100 µL of DMEM, supplemented with 10% fetal bovine serum (FBS) and 1% penicillin-streptomycin in a humid environment with 5% CO_2_ at 37°C. Following a 24 h incubation, 100 µL of the extract solutions from each sample (BM, bPVA/SIS@PDA, bPVA/SIS^+^, and bPVA/SIS^+^-NP patches) was added to the corresponding wells, displacing the pre-existing culture medium. The cells were then allowed to interact with these extract solutions over a range of incubation periods. On the 1st, 2nd, and 3rd days after incubation, the culture medium was meticulously aspirated and the wells were rinsed thrice with PBS. Subsequently, a random selection of three wells was treated with Calcein-AM (2 µM) for 20 minutes in the dark to facilitate cellular fluorescence staining, followed by microscopic examination using a fluorescence microscope (DP80, Olympus, Japan). Concurrently, another set of three wells was incubated with a 10% CCK-8 reagent for 1 h in the dark. The OD values at 450 nm were then measured using a microplate reader (Multiskan FC, Thermo Scientific, USA) to assess cellular proliferation.

The hemocompatibility of bPVA/SIS^+^-NP patch was evaluated through a hemolysis assay. Fresh rabbit blood was collected and subjected to centrifugation at 4°C at 1500 rpm for 5 minutes to sediment the erythrocytes. The cells were washed with PBS and centrifuged three times to isolate the erythrocytes. A 5% (v/v) erythrocyte suspension in PBS was then created. Deionized water served as the positive control, while PBS acted as the negative control. Both controls and the extract solutions of bPVA/SIS^+^-NP patch were each mixed with 500 µL of the 5% erythrocyte suspension and incubated at 37°C for 1 h. The mixtures were centrifuged at 1500 rpm for 5 minutes, after which the supernatants were collected for absorbance measurement at 545 nm using a microplate reader. The hemolysis ratio was calculated as follows:

$$Hemolysis rate \left( \% \right)=\frac{Ab_{test}-Ab_{nagetive}}{Ab_{positive}-Ab_{nagetive}} \times100$$

***In vitro migration analysis***

L929 cells were seeded in 6-well plates and allowed to grow for 24 h. Subsequently, a linear incision was made down the center of each well, followed by a rinse with PBS. The cells were then exposed to extract solutions of bPVA/SIS^+^-NP patch and a commercial benchmark material (BM). Photos of the cellular response were taken at 0, 12, and 24 h post-treatment.

***In vitro drug release experiment***

To assess the release profile of NP from bPVA/SIS^+^-NP patch, samples (BM patch with NP and bPVA/SIS^+^-NP patch) were immersed in 10.0 mL of PBS at 37°C on a rotary shaker (200 rpm) for over 10 d. At predetermined time points (0, 0.5, 1, 3, 5, 7, and 10 d), 1.0 mL of the release medium was withdrawn and replenished with an equal volume of fresh PBS. The NP concentration in the withdrawn medium was quantified using an NP-specific ELISA kit, and the drug release kinetics were graphically represented.

***In vivo biocompatibility***

The biocompatibility in vivo was observed by subcutaneous insertion of the patch into the back of rats. Soak bPVA/SIS^+^ in 75% alcohol for 1 h, then rinse three times in PBS medium for disinfection. Finally, 10 µL of NP (10 μg mL^‑1^) was dropped onto the SIS^+^ surface of the bPVA/SIS^+^ patch on a 48-well plate and infiltrated at 4°C for 24 h to obtain bPVA/SIS^+^-NP patch. bPVA/SIS^+^-NP and BM patches were cut into a diameter of 1cm and implanted into the back skin of each rat, for a total of three rats to repeat the experiment. Five days later, the rats were euthanized by abdominal anesthesia (3% pentobarbital sodium, 1.5 mL kg^‑1^). The subcutaneous area around bPVA/SIS^+^-NP and BM patches was excised and fixed in 4% paraformaldehyde solution for 24 h, and then sent to Wuhan Service Biotechnology Co., Ltd. for HE staining and immunohistochemical staining of CD68 and IL-6.

***Repair of the infectious abdominal wall defect in rat***

The effect of bPVA/SIS^+^-NP patch in preventing adhesion and promoting healing in infectious abdominal wall defects was assessed using a Sprague-Dawley rat model. Rats were induced under anesthesia with an intraperitoneal injection of 3% sodium pentobarbital (1.5 mL kg^‑1^). Sterile surgical procedures were followed to create a 10 mm-diameter full-thickness defect in the abdominal wall using a circular punch. The defect was inoculated with 100 μL of *E. coli* (10^7^ CFU mL^‑1^) suspension for one minute to mimic contamination, after which the excess was removed with sterile gauze. Various patches (commercial BM, SIS^+^-NP, bPVA, and bPVA/SIS^+^-NP patches) were then applied to the defects to evaluate their capacity for tissue regeneration and adhesion prevention. The patches were secured with 4-0 silk sutures, and the abdominal incisions were closed with 4-0 silk sutures.

***Morphological observation and histological evaluation***

On the 14th day after surgery, the rats were euthanized in compliance with the Animal Welfare Act. The surgical wounds were reopened to assess the abdominal adhesion and healing of the defect. Photographs were taken of the implanted patches and the healing region. Muscle tissue was dissected, fixed in a 4% paraformaldehyde solution for 24 h, and dispatched to Wuhan Servicebio Technology Co., Ltd. for histological staining, including hematoxylin and eosin (HE), Masson's trichrome, and immunohistochemical staining for CD68 and CD31. Adhesion was quantified utilizing the clinical grading of adhesion extent, adhesion type, and adhesion tenacity (Table S1, Supporting Information). The histological image was subjected to quantitative analysis using Image J software.

***Statistical analysis***

All statistical analysis was carried out using Origin 2021 software (Origin Lab Incorporation, Northampton, USA). Data were expressed as mean ± standard deviation (SD). Statistical differences between groups were determined by one-way analysis of variance (ANOVA). Statistical significance was marked with * *p* < 0.05, ** *p* < 0.01 and *** *p* < 0.001.


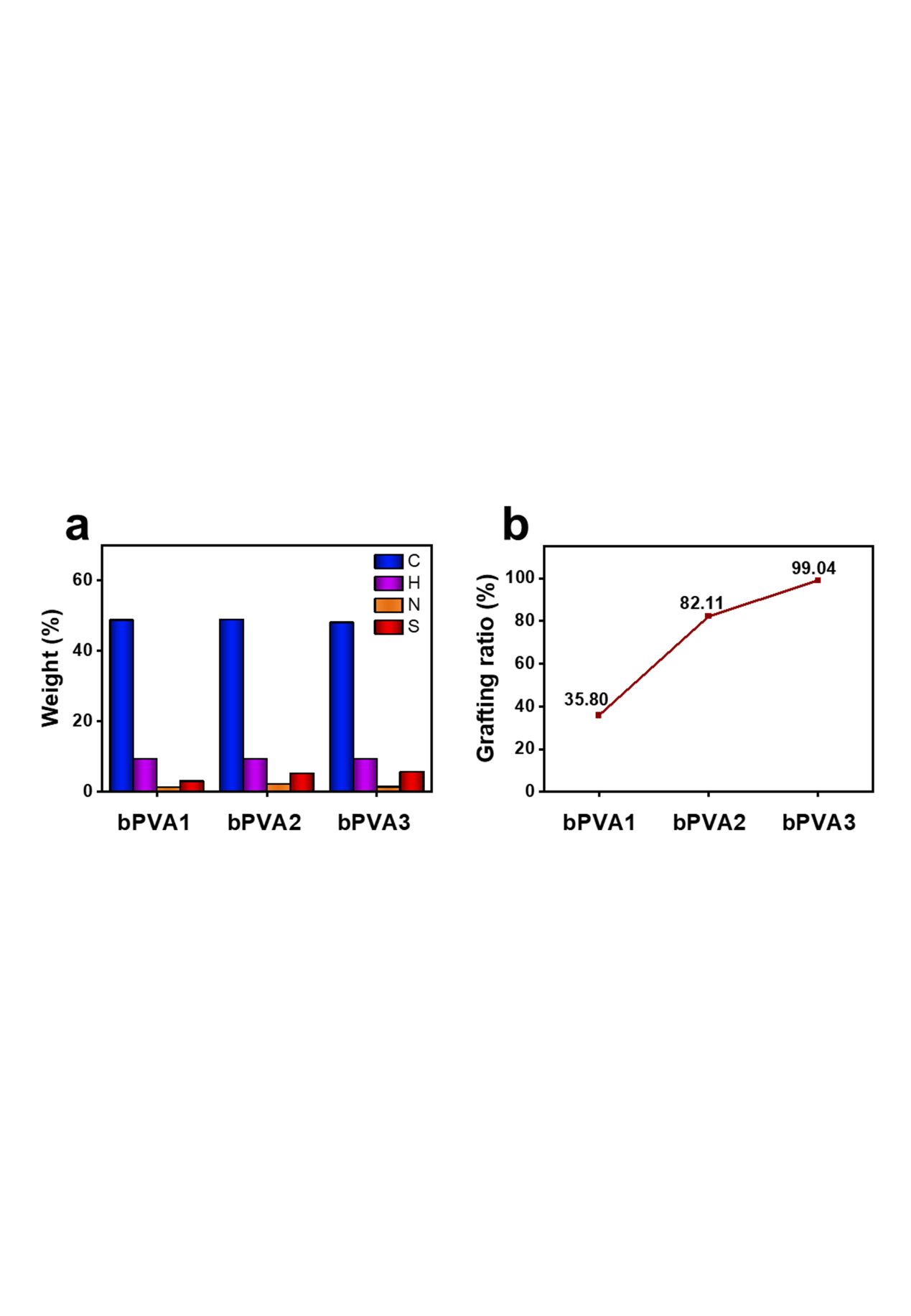


**Figure S1**. a) Elemental analysis and b) the corresponding grafting ratios of PSBMA from PVA backbone with different feeding weight ratios. Because the grafting ratio of bPVA3 doesn’t change obviously compared with bPVA2, unless otherwise specified, bPVA used in the manuscript is referred to as bPVA2.


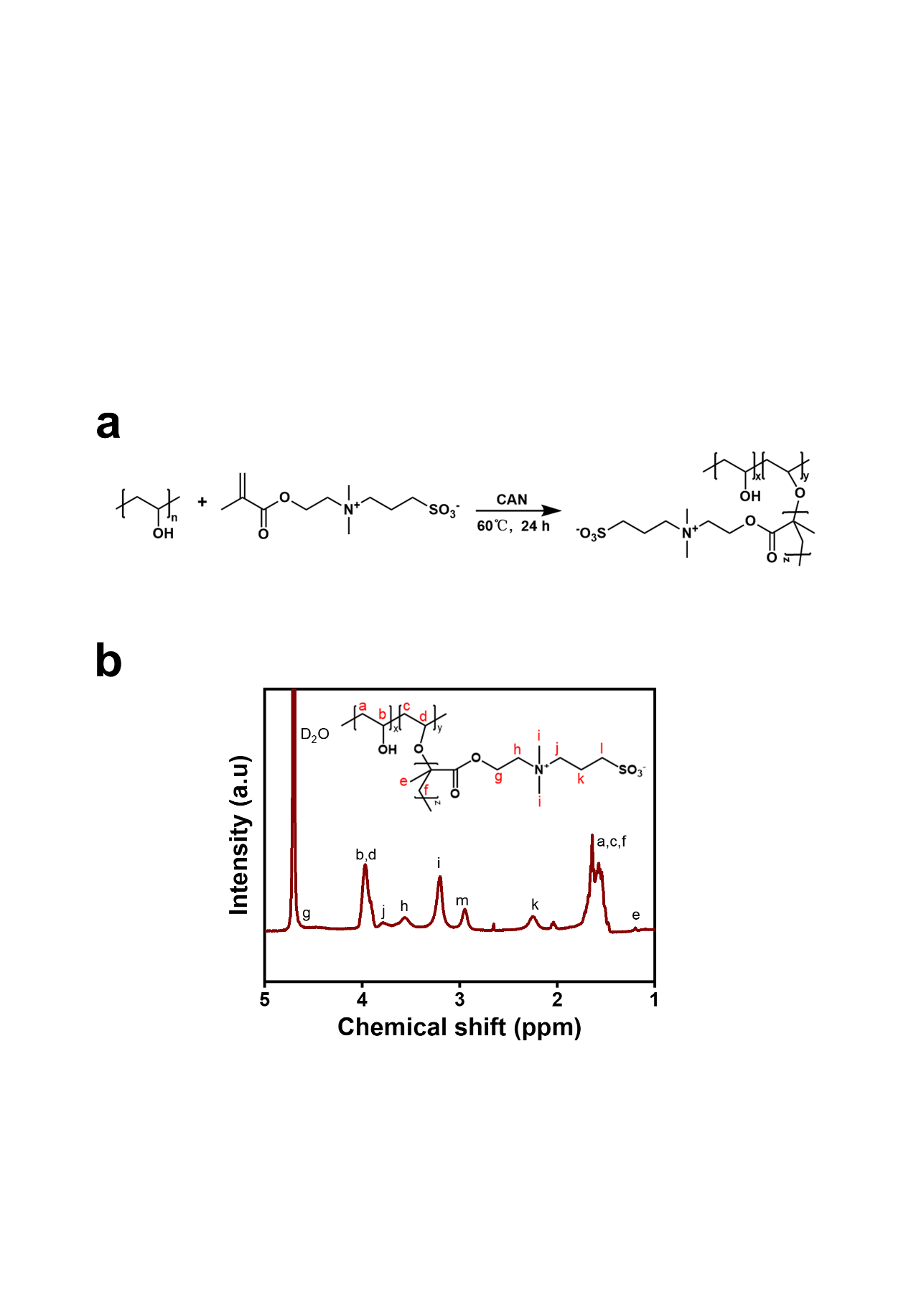


**Figure S2**. a) Synthesis of bPVA through free-radical polymerization. b) ^1^H-NMR spectrum and the corresponding proton assignment of bPVA2.


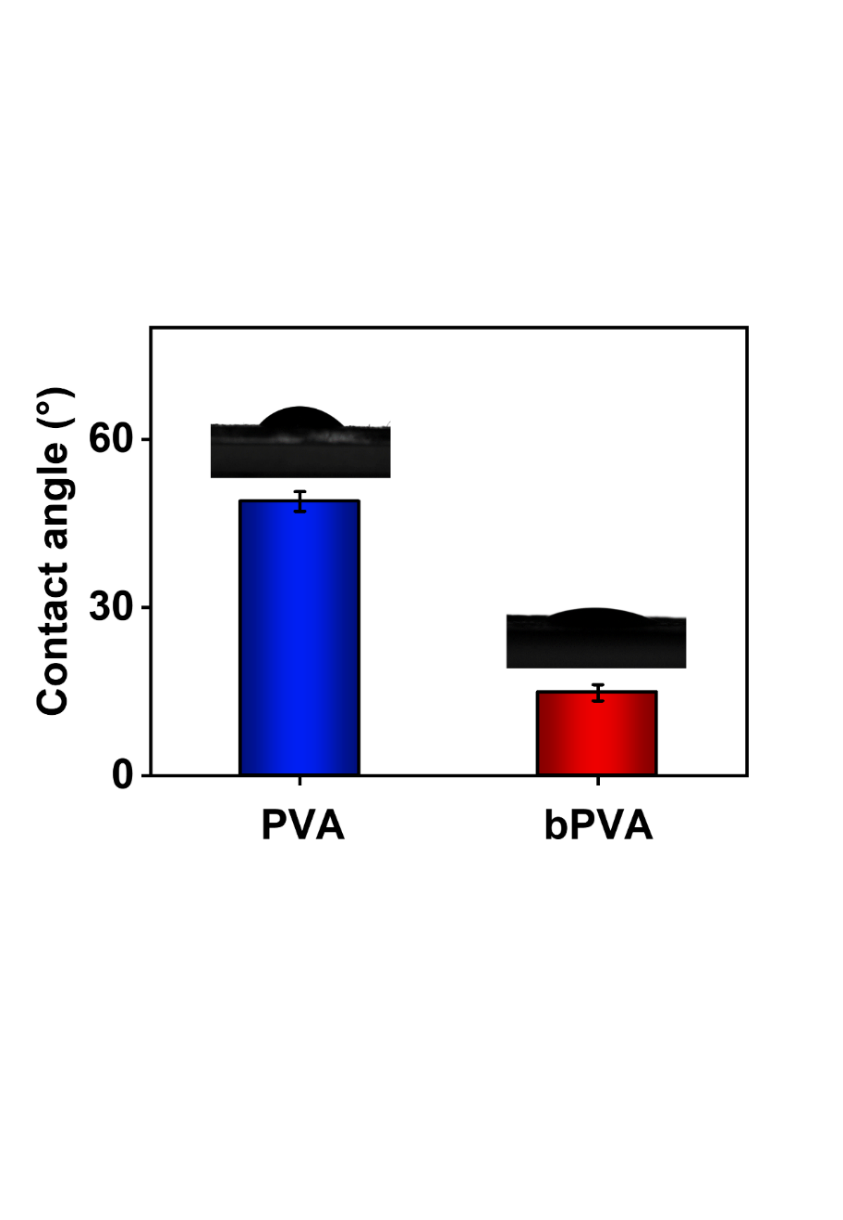


**Figure S3**. Contact angles of water on PVA and bPVA hydrogels. The data are the mean ± SD ( *n* = 3).


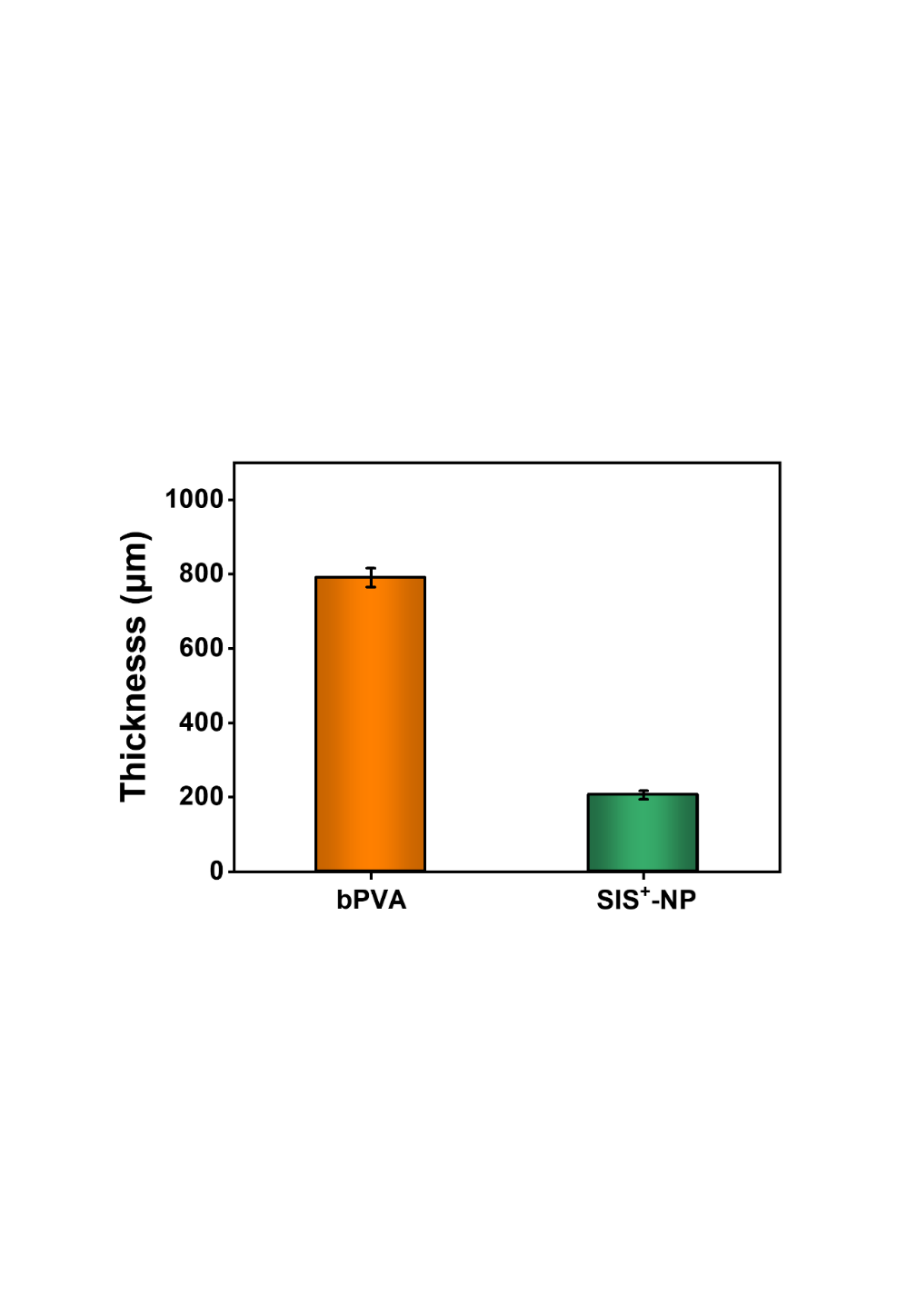


**Figure S4**. Thickness of the bPVA and SIS^+^-NP layers of bPVA/SIS^+^-NP patch. The data are the mean ± SD (*n* = 3).


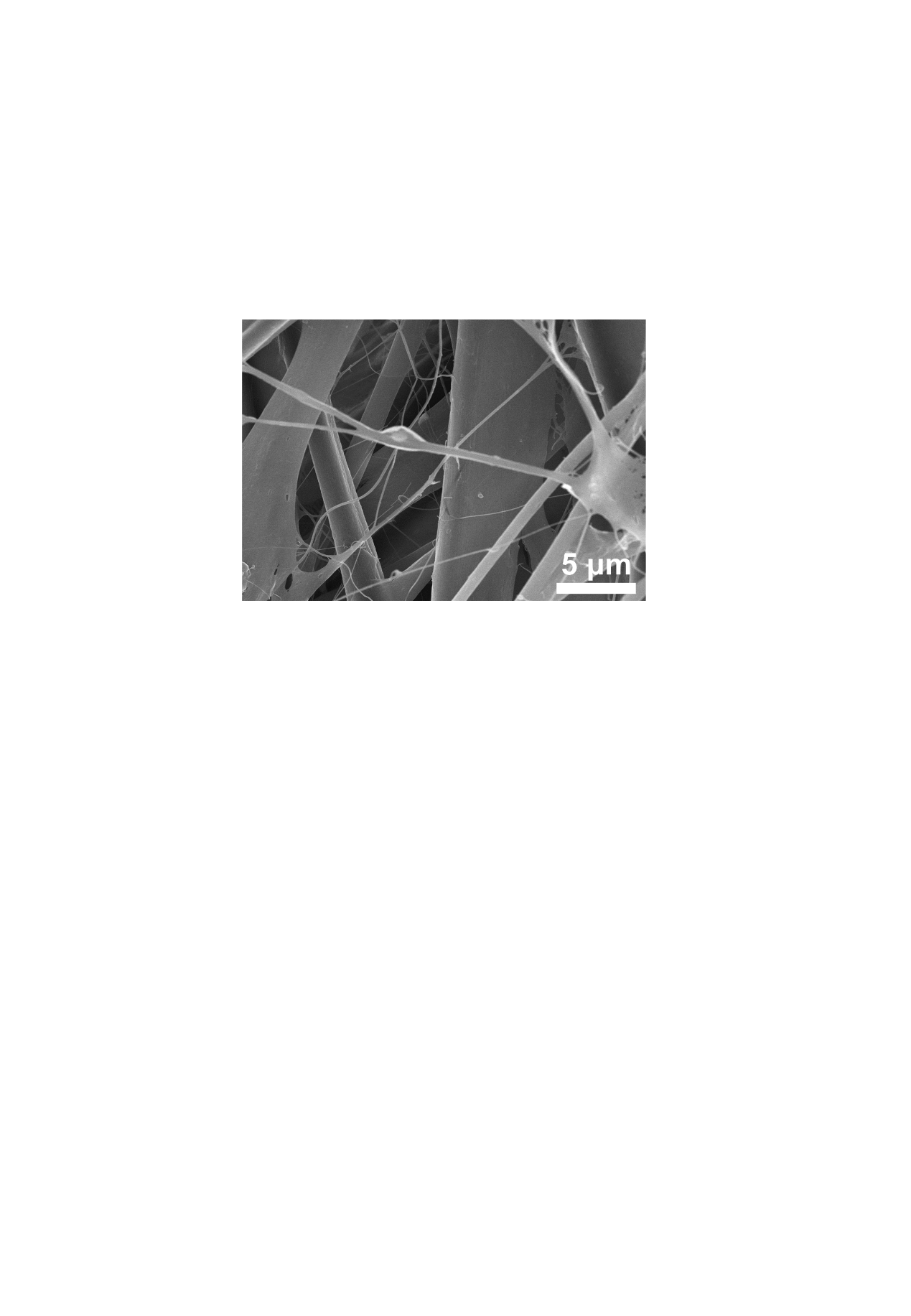


**Figure S5**. SEM image of unmodified SIS.


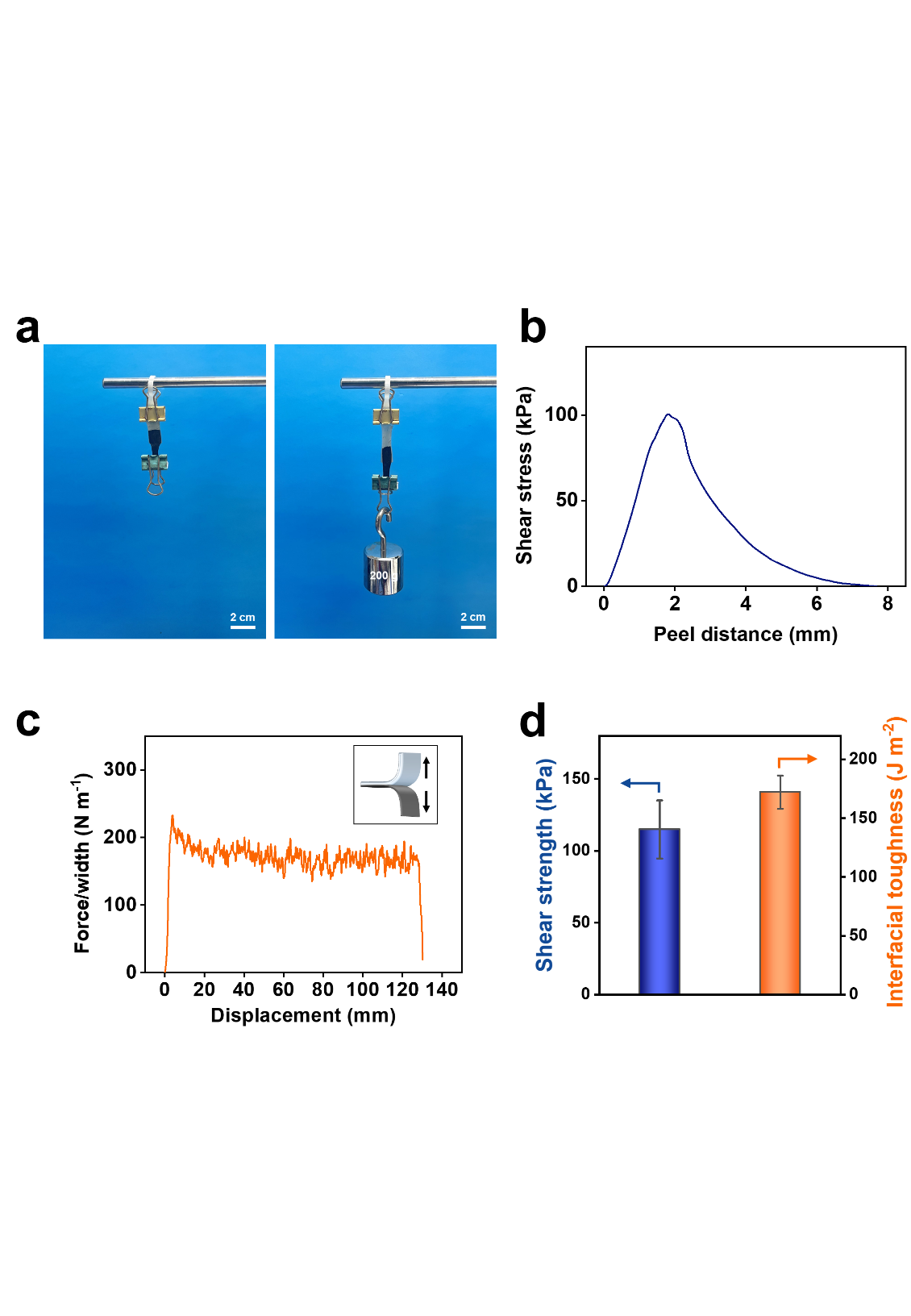


**Figure S6**. a) Digital photos of bPVA/SIS^+^-NP patch with a 200 g load for lap-shear test. b) Shear stress vs displacement curve for lap-shear test of bPVA/SIS^+^-NP patch. c) Force/width vs displacement curve for 180-degree peeling test of bPVA/SIS^+^-NP patch. d) Shear strength and interfacial toughness of bPVA/SIS^+^-NP patch. The data are the mean ± SD (*n* = 3).


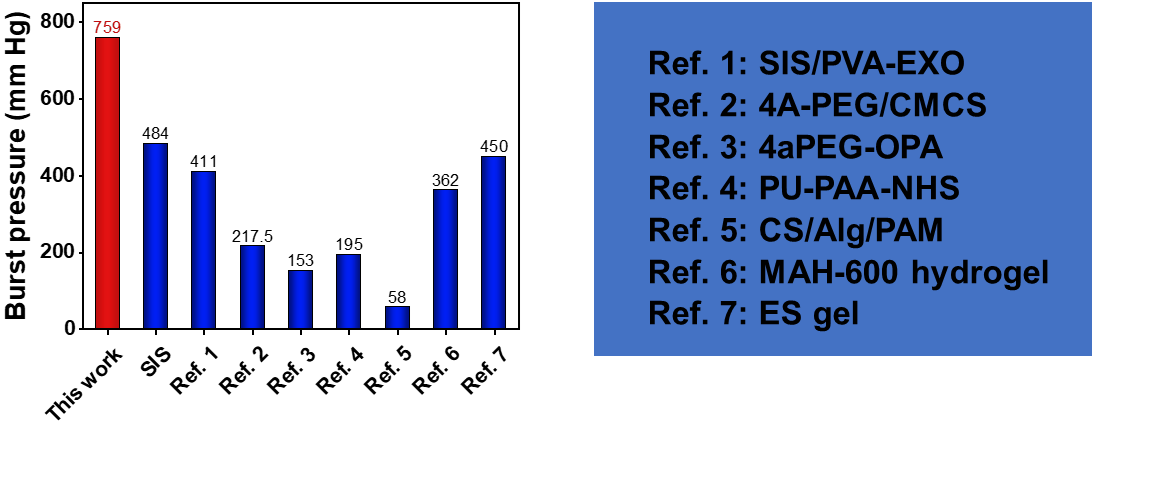


**Figure S7**. Comparison of burst pressure for bPVA/SIS^+^-NP patch and other reported anti-adhesion hydrogels^[1-7]^.


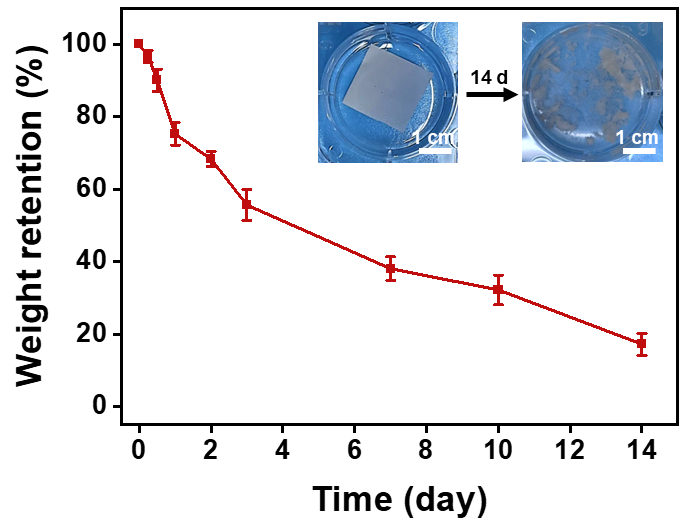


**Figure S8**. Weight retention curve and digital photos (the insets) for PHA patch in 0.2 mg mL^-1^ lipase solution.


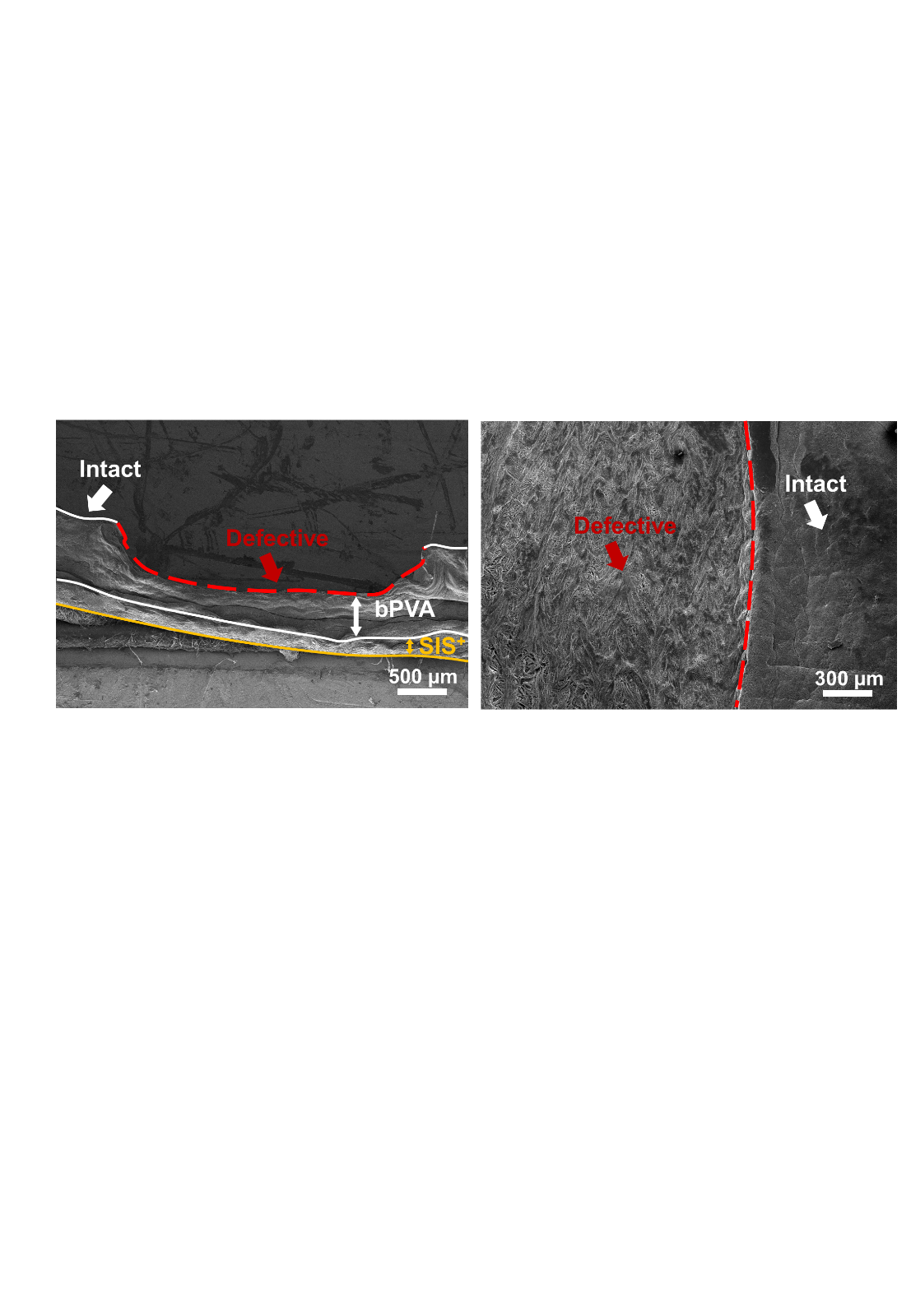


**Figure S9**. SEM images of bPVA layer with a local defect in bPVA/SIS^+^-NP patch.


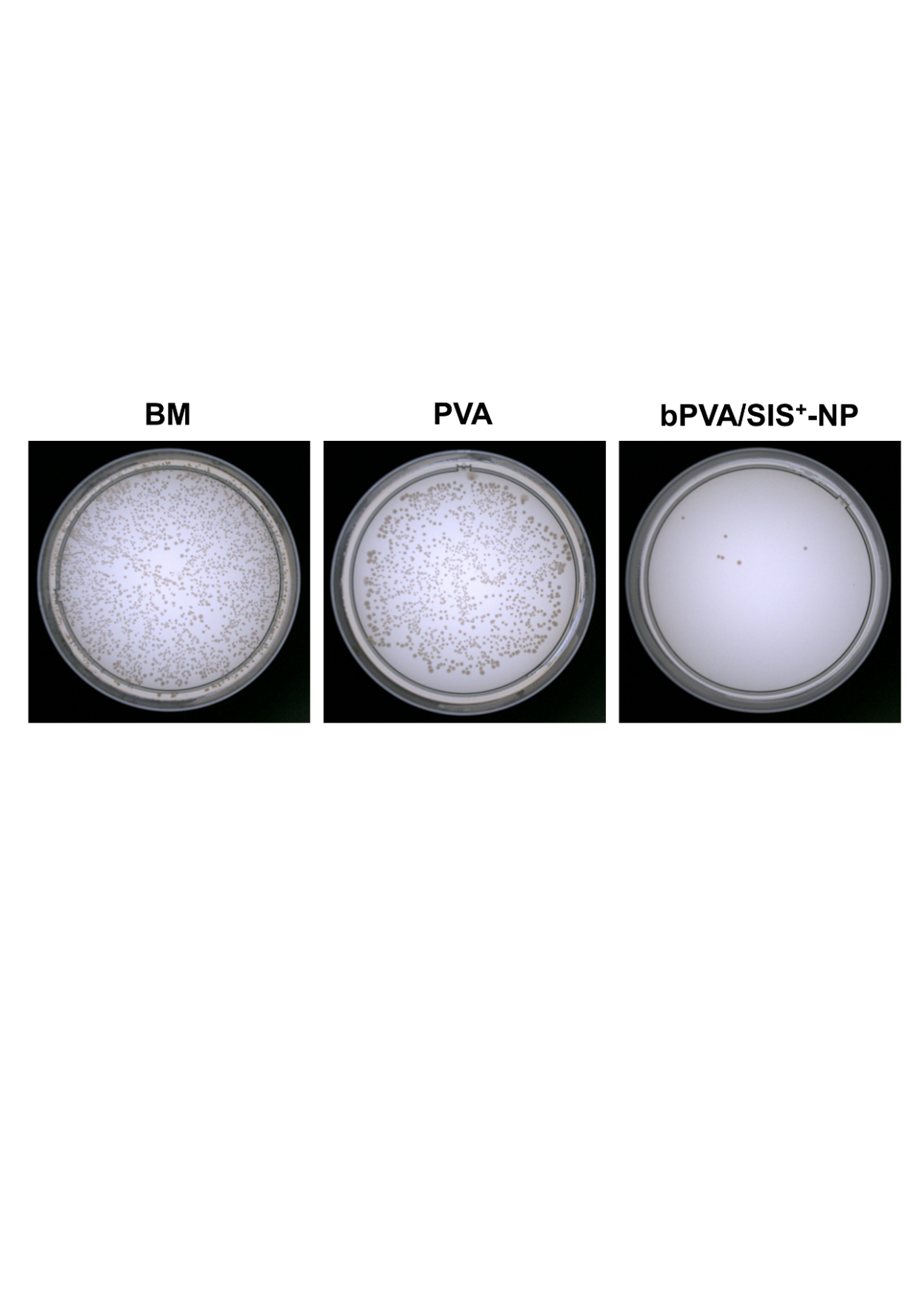


**Figure S10**. The number of *E. coli* adhering to the surface of the BM, PVA, and bPVA/SIS^+^-NP patches after implantation in subcutaneous infection wounds on the back of rats for 6 days.


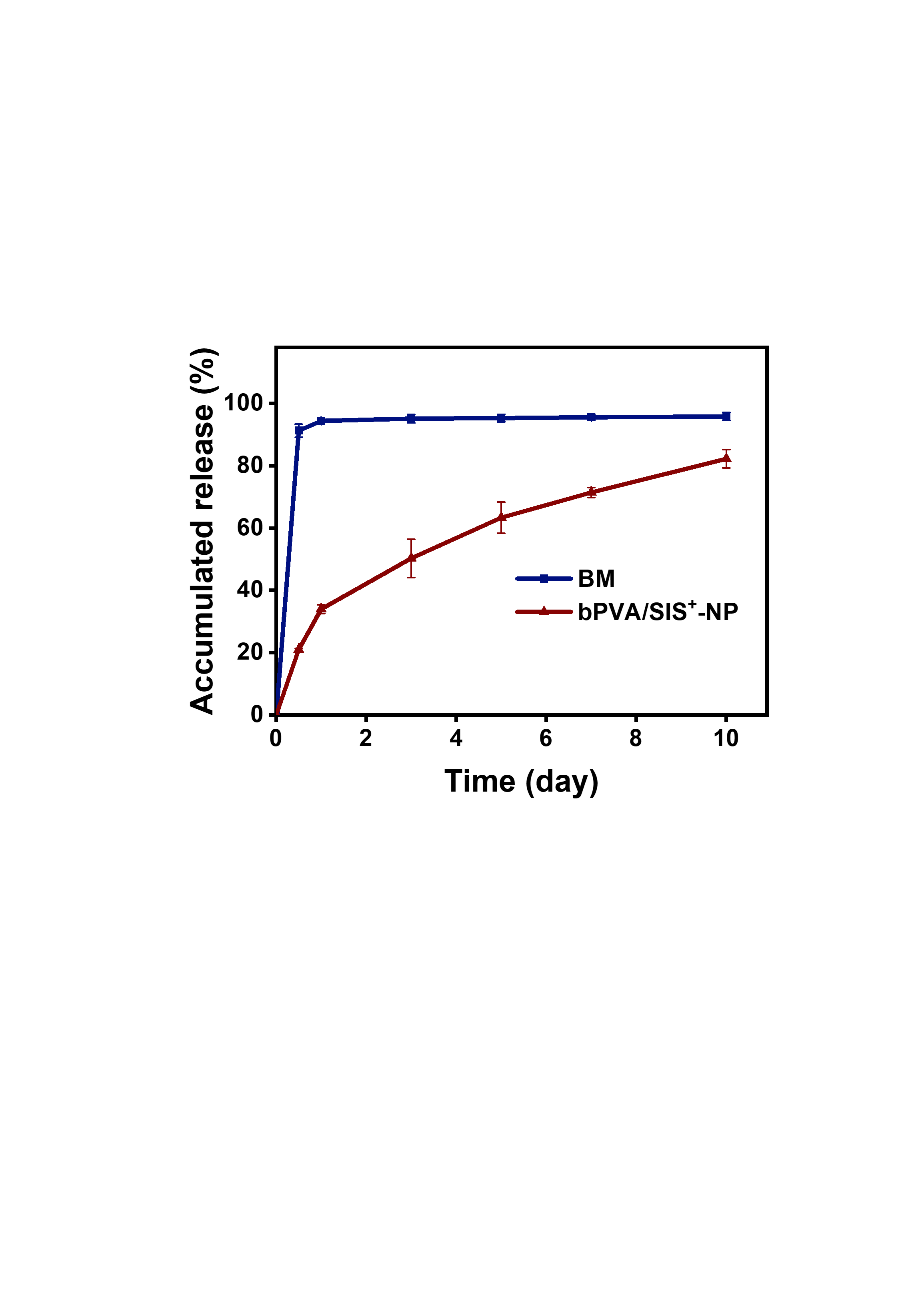


**Figure S11**. The NP release rates for BM patch with NP and bPVA/SIS^+^‑NP patch. The data are the mean ± SD (*n* = 3).


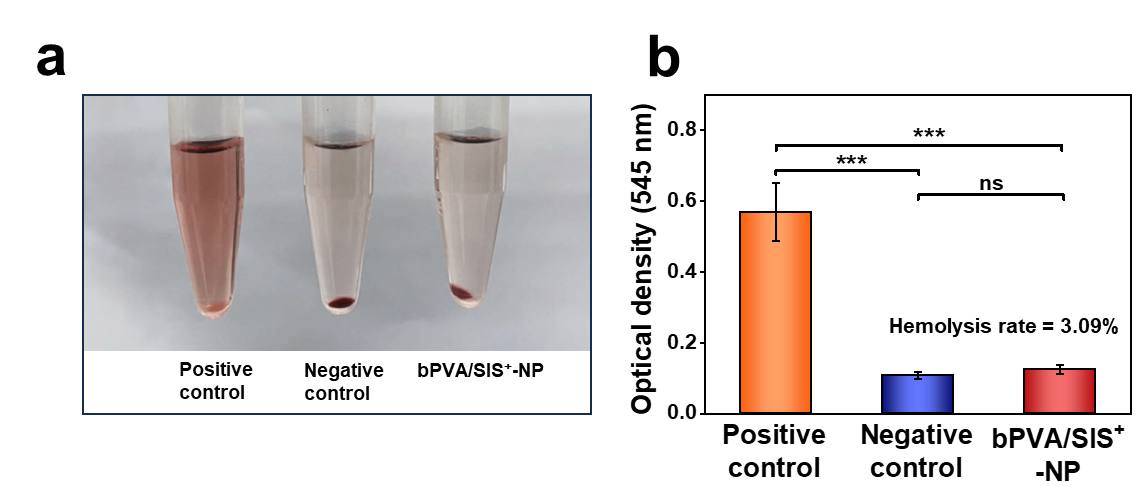


**Figure S12**. Digital photo of hemolysis assay (a) and optical density values at 545 nm for the positive control, negative control and bPVA/SIS^+^‑NP groups (b). The data are the mean ± SD (*n* = 3; *** *p* < 0.001).


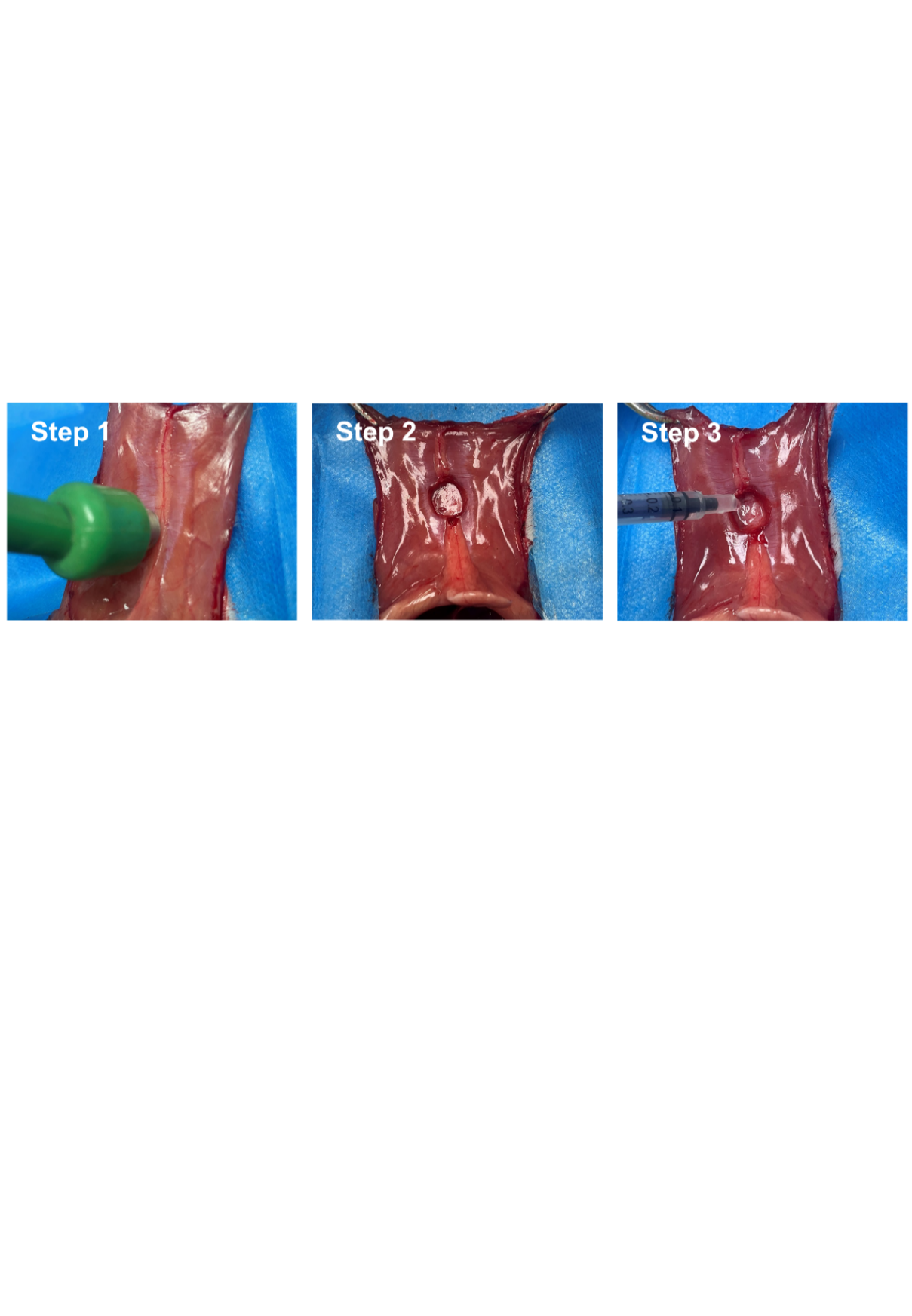


**Figure S13.** Digital photos of procedures for creating an infectious full-thickness abdominal wall defect, including removal of the peritoneum and muscular layer of the abdominal wall in rat (steps 1 and 2) and contamination with *E. coli* (step 3).

**Table S1**. Clinical adhesion scoring criteria

| Criteria ^a^ | Adhesion extent ^b^ | Adhesion type | Adhesion tenacity ^c^ |
| --- | --- | --- | --- |
| 0 | No adhesion | No adhesion | No adhesion |
| 1 | 0-25% coverage | Filmy | Easily fall apart |
| 2 | 25-50% coverage | Thick | Require traction |
| 3 | 50-75% coverage | Presence of capillaries | Require sharp dissection |
| 4 | 75-100% coverage | / | / |

^a^ Clinical adhesion score consists of adhesion extent, adhesion type, and adhesion tenacity. ^b^ The ratio of adhesion area to the total area of implant surface. ^c^ Force or surgical tools are required to detach the adhered tissues.

**References**

[1] F. Tang, D. Miao, R. Huang, B. Zheng, Y. Yu, P. Ma, B. Peng, Y. Li, H. Wang, D. Wu, *Adv. Mater.* **2024**, 36, e2307845.

[2] X. Yin, Y. Hao, Y. Lu, D. Zhang, Y. Zhao, L. Mei, K. Sui, Q. Zhou, J. Hu, *Adv. Funct. Mater.* **2021**, 31, 2105614.

[3] X. Cheng, Z. Zhang, H. Ren, Z. Zou, Y. Zhang, Y. Qu, X. Chen, J. Zhao, C. He, *Natl. Sci. Rev.* **2024**, 11, nwae160.

[4] S. J. Wu, J. Wu, S. J. Kaser, H. Roh, R. D. Shiferaw, H. Yuk, X. Zhao, *Nat. Commun.* **2024**, 15, 1215.

[5] J. Li, J. Tian, C. Li, L. Chen, Y. Zhao, *Bioact. Mater.* **2022**, 14, 389.

[6] Y. Yang, G. He, Z. Pan, K. Zhang, Y. Xian, Z. Zhu, Y. Hong, C. Zhang, D. Wu, *Adv. Mater.* **2024**, 36, e2404811.

[7] H. Wang, X. Yi, T. Liu, J. Liu, Q. Wu, Y. Ding, Z. Liu, Q. Wang, *Adv. Mater.* **2023**, 35, e2300394.
